# ﻿Six new species of *Diostracus* Loew (Diptera, Dolichopodidae) from Tibet

**DOI:** 10.3897/zookeys.1163.101533

**Published:** 2023-05-17

**Authors:** Yajun Zhu, Chufei Tang, Ding Yang

**Affiliations:** 1 Technical Center for Animal, Plant and Food Inspection and Quarantine of Shanghai Customs, Shanghai 200135, China Technical Center for Animal, Plant and Food Inspection and Quarantine of Shanghai Customs Shanghai China; 2 Institute of Leisure Agriculture, Jiangsu Academy of Agricultural Sciences, Nanjing 210014, China Institute of Leisure Agriculture, Jiangsu Academy of Agricultural Sciences Nanjing China; 3 Department of Entomology, China Agricultural University, Beijing 100193, China China Agricultural University Beijing China

**Keywords:** Key, long-legged fly, morphology, The Himalayan region

## Abstract

Six species of *Diostracus* from Tibet are described as new to science: *D.concavus***sp. nov.**, *D.fasciculatus***sp. nov.**, *D.laetus***sp. nov.**, *D.polytrichus***sp. nov.**, *D.strenus***sp. nov.**, and *D.translucidus***sp. nov.** A key to the species from Tibet of the genus is provided. The distribution of the genus in Tibet is also discussed.

## ﻿Introduction

*Diostracus* belongs to the subfamily Hydrophorinae of Dolichopodidae. These flies are usually stout and larger than other dolichopod flies. They prefer to live on the vertical or oblique surfaces of rocks at altitudes 1000 m to 3500 m, with slow water flow, or a thin water layer on the surface, or just wet, but they do not like those rocks behind streams or waterfalls.

Before our study, 101 species of the genus had been reported (Zhu 2006; Zhu et al. 2007a, b; Grichanov 2013, 2015, 2017; Pusch 2014; Wang et al. 2015). The first *Diostracus*, *D.prasinus* Loew, was named in 1861 from the Nearctic Region, whereas most species of the genus were reported from the Palaearctic and Oriental realms. Remarkably, *Diostracus* shows great diversity in the Himalayas, which is the junctions of the two realms, and 39 species have been reported from this area (Zhu 2006; Wang et al. 2015). Tibet is located in the east of the Himalaya Mountains. However, there were only three species of *Diostracus* known from Tibet: *D.nebulosus* Takagi, 1972 is widely distributed in the Himalayas, while *D.acutatus*Wang et al., 2015, and *D.tibetensis*Wang et al., 2015 were reported in Nyingchi, Tibet.

Here we provide an investigation of the diversity of *Diostracus* in Tibet and six new species are reported.

## ﻿Materials and methods

This work is based on the material collected by sweep netting from Tibet in 2013 and 2018. The main locality is Yadong County (88°52'–89°30'E, 27°23'–28°18'N), located on the southern slope of the Himalaya Mountains. All the altitudes of localities are approximately 3000 m a.s.l. The specimens are deposited in the Entomological Museum of China Agricultural University, Beijing (**CAU**). The information about the specimens studied in the paper are presented in Table 1. Morphological terminology for adult structures mainly follows Cumming and Wood (2017).

**Table 1.** List of materials studied in this paper.

Species name	Number and sex	Locality	Altitude	Geographical coordinates	Type or other material
***Diostracusacutatus***	3 ♂♂ 6 ♀♀	Tibet, Shigatse, Yatung County	2700–3200 m	27°48'N, 88°90'E	Other material
	2 ♂♂ 1♀	Tibet, Shigatse, Yatung County, Pamaimang	ca 3350 m	27°48'N, 88°90'E	Other material
***D.concavus***	1♂	Tibet, Bomi, Gagela Mt.	3026 m	/	Holotype
***D.fasciculatus***	1♂	Tibet, Shigatse, Yatung County	2700–3200 m	27°48'N, 88°90'E	Holotype
	3 ♂♂ 2 ♀♀	Tibet, Shigatse, Yatung County	2700–3200 m	27°48'N, 88°90'E	Paratypes
	11♂♂ 4 ♀♀	Tibet, Shigatse, Yatung County, Pamaimang	ca 3350 m	27°48'N, 88°90'E	
***D.laetus***	1♂	Tibet, Shigatse, Yatung County	2700–3200 m	27°48'N, 88°90'E	Holotype
***D.polytrichus***	1♂	Tibet, Shigatse, Yatung County, Pamaimang	ca 3350 m	27°48'N, 88°90'E	Holotype
	2 ♂♂ 2 ♀♀	Tibet, Shigatse, Yatung County, Pamaimang	ca 3350 m	27°48'N, 88°90'E	Paratypes
***D.strenus***	1♂	Tibet, Shigatse, Yatung County	2700–3200 m	27°48'N, 88°90'E	Holotype
	1 ♂	Tibet, Shigatse, Yatung County, Pamaimang	ca 3350 m	27°48'N, 88°90'E	Paratype
***D.translucidus***	1♂	Tibet, Medog	/	/	Holotype
	2 ♀♀	Tibet, Medog	/	/	Paratypes

The following abbreviations are used:

**acr** acrostichal,

**ad** anterodorsal bristle (s),

**av** anteroventral bristle (s),

**dc** dorsocentral bristle (s),

**CI** fore coxa,

**CII** mid coxa,

**CIII** hind coxa,

**FI** fore femur,

**FII** mid femur,

**FIII** hind femur,

**It** fore tarsomeres,

**h** humeral bristle,

**IIt** mid tarsomeres,

**IIIt** hind tarsomeres,

**LI** fore leg,

**MSSC** male secondary sexual characters,

**pvt** postrovertical bristle (s),

**npl** notopleural bristle (s),

**oc** ocellar bristle(s),

**pd** posterodorsal bristle(s),

**ph** postohumeral bristle,

**psa** postosupraalar bristle,

**pv** postoventral bristle(s),

**TI** fore tibia,

**TII** mid tibia,

**TIII** hind tibia,

**sa** supraalar bristle,

**sc** scutellar bristle(s),

**t** tarsomeres,

**vt** vertical bristle(s).

Each holotype male was submitted to barcode sequencing, using the primers LCO1480/ HCO2198 (Folmer et al. 1994), under the following experimental procedures: 3 min at 95 °C for the first cycle, reactions were amplified through 35 cycles at following paraments, 30 s at 95 °C, 30 s at 55 °C, and 90 s at 72 °C, then elongation for 1 cycle at 72 °C for 10 min. The sequences were uploaded to GenBank (Table 2). Females were also sequenced to pair them to males, in addition to examination of their morphological characters.

**Table 2.** Barcode label data for *Diostracus* species reported in this paper.

Species	Specimen catalog code	Sex	GenBank code	GenSeq
***D.acutatus***	Di03M	male	MT447459	Genseq-5 COI
	Di03F	female	MT462596	Genseq-5 COI
***D.concavus***	Di06M	male	MT452300	Genseq-1 COI
***D.fasciculatus***	*Diostracus* sp1	male	MT080656	Genseq-2 COI
	Di01F	female	MT462594	Genseq-2 COI
***D.laetus***	Di05M	male	MT452307	Genseq-1 COI
***D.polytrichus***	Di02M	male	MT438694	Genseq-2 COI
	Di02F	female	MT462595	Genseq-2 COI
***D.strenus***	Di04M	male	MT447458	Genseq-2 COI
***D.translucidus***	*Diostracus* sp7 male	male	OP249496	Genseq-1 COI
	*Diostracus* sp7 female	female	OP249495	Genseq-2 COI

## ﻿Taxonomic accounts

### ﻿Genus Diostracus

Taxon classificationAnimaliaDipteraDolichopodidae

Loew, 1861

10714B10-3EB5-5536-A8D3-D4D443EF0F7D

Diostracus Loew, 1861: 44. Type species: Diostracusprasinus Loew, 1861 (monotypy).
Sphyrotarsus Mik, 1874: 342. Type species: Sphyrotarsusargyrostomus Mik, 1874 (monotypy).
Asphyrotarsus Oldenberg, 1916: 193. Type species: Liancalusleucostomus Loew, 1861 (original designation).
Takagia Negrobov, 1973: 1520 (as subgenus of Sphyrotarsus Mik, 1874) (not Matsumura, 1942). — Negrobov 1978: pl. CLXI (as genus, in error). Type species: Sphyrotarsusstackelbergi Negrobov, 1965 (original designation).
Lagodechia Negrobov & Tsurikov, 1996: 632. Type species: Diostracusspinulifer Negrobov & Tsurikov, 1988 (monotypy).
Ozmena Özdikmen, 2010: 265 (new name for Takagia Negrobov, 1973, not Matsumura, 1942) (as subgenus of Sphyrotarsus Mik, 1874).

#### Diagnosis.

Medium to huge dolichopodid flies (males body length 3.4–7.6 mm, usually larger in females). Body stout, metallic green, always with pollinosity. Vertex weakly concave; upper occiput slightly concave. Scape with or without dorsal seta; arista subapical or sub-basal. Palpus rather large and loosely applied on proboscis, sometimes elongated, beyond the apex of proboscis in males, and relatively smaller in females. Proboscis bulky. Acr absent; four or six pairs of dc; one or two strong npl; scutellum with two strong sc, sometimes with marginal hairs. Crossvein m-cu longer than final section of 5^th^ longitudinal vein. Legs and wings are often modified in males, which are usually the identical characters for groups or species. Abdomen cylindrical, with five visible segments; Sternite I or IV sometimes with produced process, and Sternite V usually split into pair of sclerites in males.

### ﻿Key to species (males) of *Diostracus* from Tibet

**Table:** 

1	Palpus normal, not reaching apex of proboscis; scutellum with pair of sc and four or five pairs of marginal hairs; CI with row of anterior hairs and two strong recurved spines at extreme apex; FI with a deep hollow at base; wing indistinctly tinged grayish, apically with three translucent windows; FII with row of dense ad on apical 2/5	***D* . *translucidus* sp. nov.**
–	Palpus prolonged, reaching apex of proboscis; scutellum with pair of sc, without marginal hairs; other characters variable	**2**
2	Empodium and pulvilli reduced into minute protuberance (*fenestratus* group)	**5**
–	Empodium and pulvilli distinct	**3**
3	Wing with a dark square marking on vein M near crossvein; five dc	***D* . *nebulosus* Takagi**
–	Wing with a small round black nodule at middle of crossvein; six dc	**4**
4	Cercus finger-like, straight, with long yellow hairs	***D* . *tibetensis* Wang et al.**
–	Cercus lamellate with broad basal half	***D* . *polytrichus* sp. nov.**
5	Wing with dark and yellow markings at middle; discal crossvein strongly sinuate, S-shaped; anterodistal corner of discal cell with an accessory cellula (*pulchripennis* subgroup)	***D* . *laetus* sp. nov.**
–	Wing without distinct marking; discal crossvein nearly straight; anterodistal corner of discal cell without accessory cellula (*flex* subgroup)	**6**
6	Posterior margin of wing somewhat prolonged along vein CuA_1_; apex of TII swollen with two rows of narrow flat willow leaf-like ventral bristles, row of long av (anterior ones somewhat curved), rows of pale long ventral hairs (2–3 × longer than TII depth, curved), row of erect pv along whole length (as long as TII depth), apically with three long bristles	***D* . *fasciculatus* sp. nov.**
–	Posterior margin of wing normal; TII normal, not swollen	**7**
7	Crossvein m-cu elongated, strongly bent, margined with black on long anterior portion, and with blackish spot at short posterior portion; FI slightly thickened; It_1_ with acute apicoventral corner, It_2_ with an acute ventral process near extreme base; abdominal sternite I with a nearly acute process at middle; sternite IV medially with an obtuse anterior process and two short thin, contiguous posterior processes bearing bundle of brown hairs	***D.acutatus* Wang et al.**
–	Crossvein m-cu not elongated, acutely and deeply arched to vein M_1_, forming a ‘h’-shaped curve, with a jet-black brand inside curve; FI distinctly thickened; It_1_ shortened, without acute apicoventral corner; abdominal sternite without such appendage	**8**
8	Propleuron with two or three sparse short pale hairs on upper portion and one or two short pale hairs on lower portion; It_3–5_ normal, without ventral suture	***D.strenus* sp. nov.**
–	Propleuron with group of seven long pale hairs on upper portion and group of nearly 20 long pale hairs on lower portion; It_3–5_ with a ventral suture	***D* . *concavus* sp. nov.**

### ﻿ Diostracus acutatus

Taxon classificationAnimaliaDipteraDolichopodidae

Wang, Wang & Yang, 2015

EA933270-0073-57E2-AFFD-F4C7AA46E373

**Figure 1.**
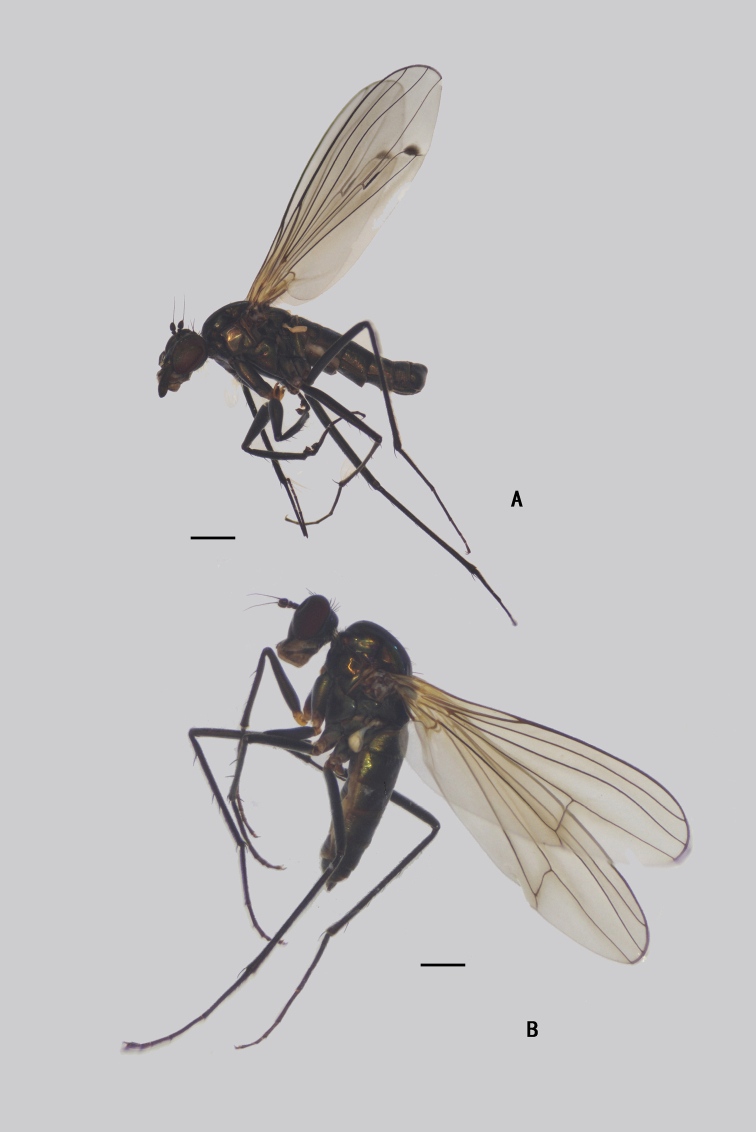
*Diostracusacutatus***A** male; lateral view **B** female, lateral view. Scale bars: 1 mm.

**Figure 2.**
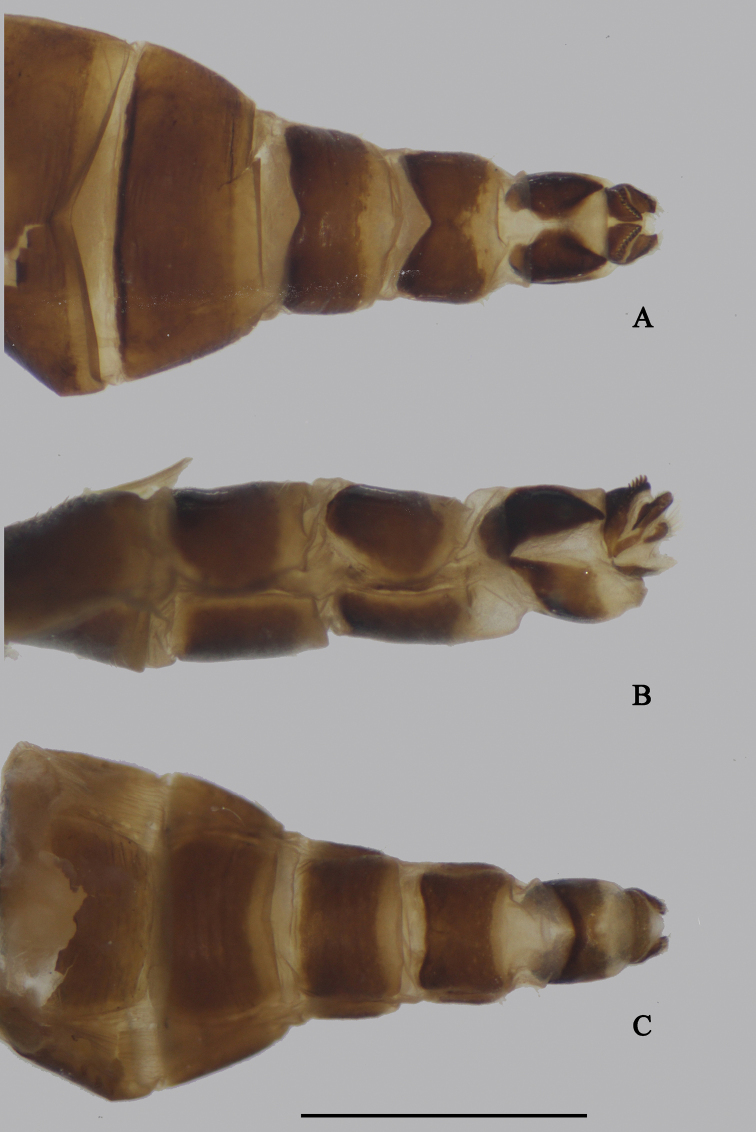
*D.acutatus* female, abdomen **A** dorsal view **B** lateral view **C** ventral view. Scale bar: 1 mm.

Figs 1 , 2 , 31A, B , 32A, B

Diostracus acutatus Wang, Wang & Yang, 2015: 96, figs 1–6.

#### Material examined.

China • 3 ♂♂ 6 ♀♀, Tibet, Shigatse, Yatung County; 27°48'N, 88°90'E; 13 VII. 2018; 2700–3200 m; leg. Yajun Zhu; • 2 ♂♂ 1♀, same data as for preceding; Pamaimang; 14 VII. 2018; ca 3350 m.

#### Diagnosis.

The species belongs to the *fenestratus* group, characterized by specialized It_1_ and It_2_ (MSSC). It_1_ distinctly shortened, with a nearly acute apicoventral process; It_2_ basally bent with a short finger-like ventral process near extreme base. Females of *D.acutatus* are characterized by the apical or subapical antenna, oblique crossvein, m-cu oblique, and brownish trochanters.

**Male** (Fig. 1A). Same as description of Wang et al. (2015).

**Female** (Fig. 1B). Body length 6.0–6.4 mm; wing length 6.9–7.8 mm. Antennal scape with two short dorsal bristles, first flagellomere somewhat prolonged, 1.2 × longer than width, arista apical or subapical, 4.6 × longer than first flagellomere (Fig. 31A, B). Proboscis yellowish brown with blackish edge; palpus relatively smaller than males, not reaching apex of proboscis. six dc, anterior four short and weak, 5^th^ long and weak. Propleuron with a single pale hair on upper portion, and group of 5–7 long pale hairs on lower portion. Legs black, except trochanters yellowish brown. CI without distinctive bristle or hair, but with short pale anterior hairs on lower portion; FI without distinct bristles; TI with an ad at basal 1/4, four pd, apically with one bristle and comb of anterior bristles; FII with two anterior bristles on apical 1/3; TII with three ad, two pd, apically with five bristles; FIII with three anterior bristles on apical 1/3; TIII with four ad, two pd, 4–6 weak v, apically with two bristles. Wing (Fig. 32A, B): m-cu somewhat curved, forming right angle with CuA_1_. Halter yellow.

***Female terminalia*** (Fig. 2): Abdominal tergite VIII split into pair of sclerites; epiproct split into pair of triangular hemitergites, apically with row of seven strong curved spines; dorsal lobes of cercus rounded in lateral view, with yellow bristles; ventral lobes of cercus membranous.

#### Remarks.

*Diostracusacutatus* is similar to *D.nishidai* Saigusa, in that they both have acute apico-ventral corners of It_1_ and It_2_ and the shapes of wings and the appendages on abdominal sternite IV are nearly identical. But for males, they are different in the shapes of the main lobe of surstylus, and the apicoventral corner of It_1_ in *D.acutatus* is sharper.

### ﻿ Diostracus concavus sp. nov.

Taxon classificationAnimaliaDipteraDolichopodidae

426B2DFC-6A54-5C9B-BA7B-F06E961436F1

https://zoobank.org/916C3BDC-2FA0-4862-807F-B799C54D4A06

Figs 3 , 4 , 5 , 6

#### Type material.

***Holotype***: China • ♂, Tibet, Nyingchi, Bomi, Gagela Mountain, 3026 m, 2013. VII. 13, leg. Xiaoyan Liu.

#### Diagnosis.

MSSC: first flagellomere 1.5 × longer than wide; propleuron with group of seven long pale hairs on upper portion and group of ~ 20 long pale hairs on lower portion; It_1_ shortened and expanded, concave ventrally, forming a hollow with an expanded It_2_. Wing (Fig. 4B) hyaline, m-cu acutely and deeply arched to vein M_1_, forming an ‘h’-shaped hairpin with a slender jet-black mark inside.

#### Description.

**Figure 3.**
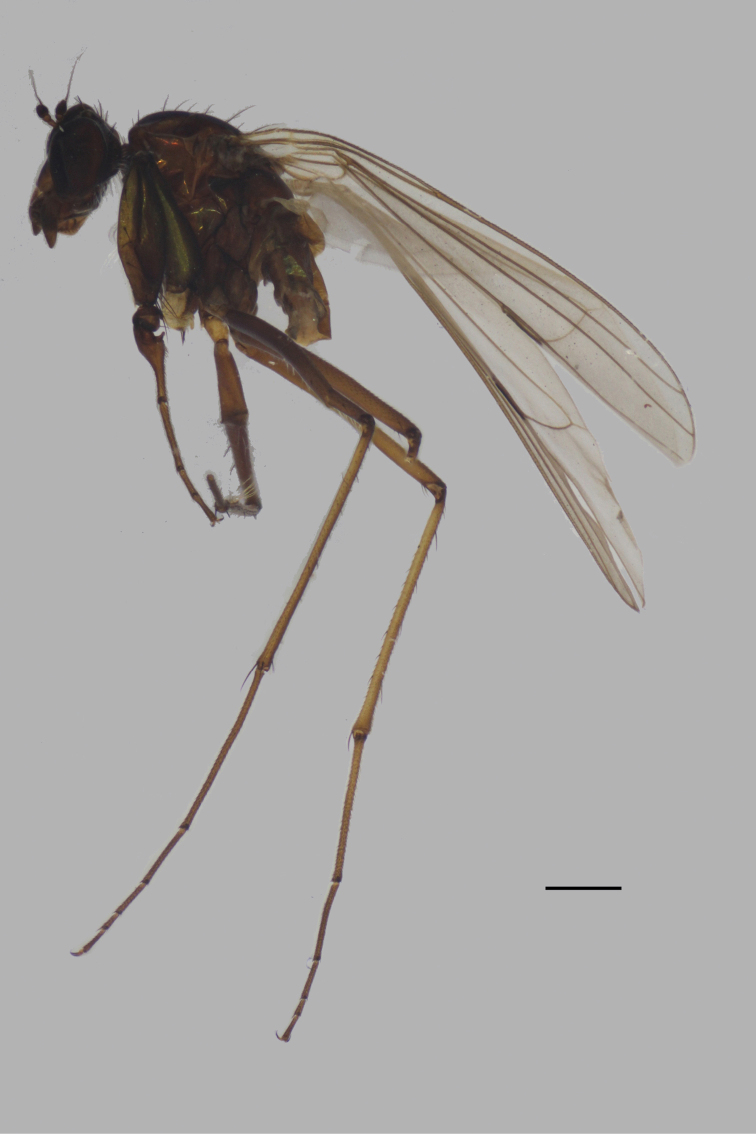
*D.concavus*, male; lateral view. Scale bar: 1 mm.

**Male** (Fig. 3). Body length 5.7 mm; wing length 6.8 mm.

**Figure 4.**
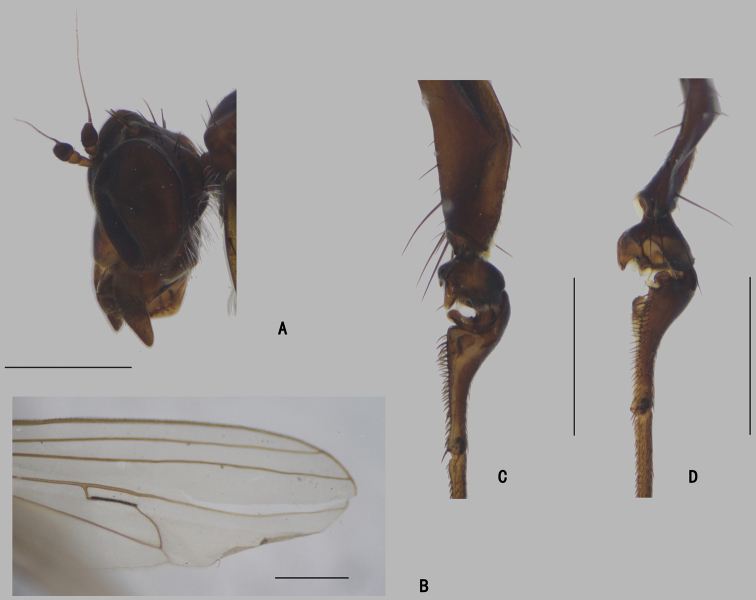
*D.concavus* male **A** head, lateral view **B** apex of wing **C** apex of TI and It_1-2_, posterior view **D** apex of FITI and It_1-2_, anterior view. Scale bars: 1 mm.

***Head*** (Fig. 4A) dark metallic green with pale gray pollinosity. Eyes separated; face widened towards clypeus. Hairs and bristles on head black; lower postocular bristles including posteroventral hairs pale. Ocellar tubercle distinct, with pair of strong oc, without posterior hairs; vt short, 0.7 × as long as oc, nearly as long as pvt. Antenna black; scape without dorsal bristle; first flagellomere subtriangular, 1.5 × longer than wide; arista subapical, 5.2 × as long as first flagellomere, nearly bare. Proboscis blackish with pale hairs; palpus lobate, 3 × as long as broad, blackish with a purple luster, without distinctive bristle.

***Thorax*** dark metallic green with pale gray pollinosity. Hairs and bristles on thorax black; six mostly hair-like dc except posterior most one dc longest and thick; acr absent; two h, one ph, two npl, one sa, one psa; scutellum with pair of long sc. Propleuron with group of seven long pale hairs on upper portion and group of nearly 20 long pale hairs on lower portion.

***Legs*** nearly entirely black except fore trochanter dark yellow; claws well developed, empodium and pulvilli reduced. Hairs and bristles on legs black except those on coxae pale. CI with cluster of anterior dense, erect, long, pale hairs on apical 1/4 (nearly as long as CI) and comb of pale hairs along anterior margin; CII and CIII nearly bare. Fore trochanter with rows of tiny ventral spines and a hook-like posterior process. FI distinctly thickened, with cluster of four or five erect ventral bristles at extreme base; TI distinctly thickened, weakly curved, with 3 ad, two pd, row of six long pv on apical 1/4; It_1_ shortened and expanded, concave ventrally, forming a hollow with expanded It_2_, anterior margin expanded into two dentiform lobes with row of four or five bristles, ventral margin expanded into a lobe at base (corresponding to the lobe of It_2_), with a subapical pv (Fig. 4C, D); basal half of It_2_ expanded and concave ventrally, anterior margin expanded into a pale dentiform lobate and a rectangular lobate, posterior ventral margin expanded into a finger-like lobe at extreme base, apical half with rows of erect ventral bristles, apical half of It_2_, It_3–5_ with a ventral suture. FII somewhat flattened laterally, with three ad on apical 1/3; TII with three weak ad, three weak pd, apical 1/5 with two rows of long pale anteroventral hairs (longest ones nearly as long as 1/4 of TII) and row of erect short pv (nearly as long as TII depth), and row of long pale posteroventral hairs along whole length (nearly as long as FII depth), apically with three long bristles. FIII with an anterior bristle and two curved av subapically; TIII with four ad and four pd, apically with two strong long bristles. Relative lengths of tibia and five tarsomeres: LI 5.0: 0.7: 2.5: 2.0: 1.0: 1.3; LII 9.0: 6.1: 2.2: 1.3: 0.7: 1.0; LIII 10.3: 5.2: 3.0: 1.5: 0.7: 1.0.

***Wing*** (Fig. 4B) hyaline, indistinctly tinged grayish; veins dark brown; crossvein m-cu not elongated, acutely and deeply arched to vein M_1_, forming a ‘h’-shaped curve with M_1_, with a slender jet-black brand inside hairpin curve; posterior margin somewhat prolonged along vein CuA_1_. Squama brown with brown hairs. Halter pale (somewhat faded).

**Figure 5.**
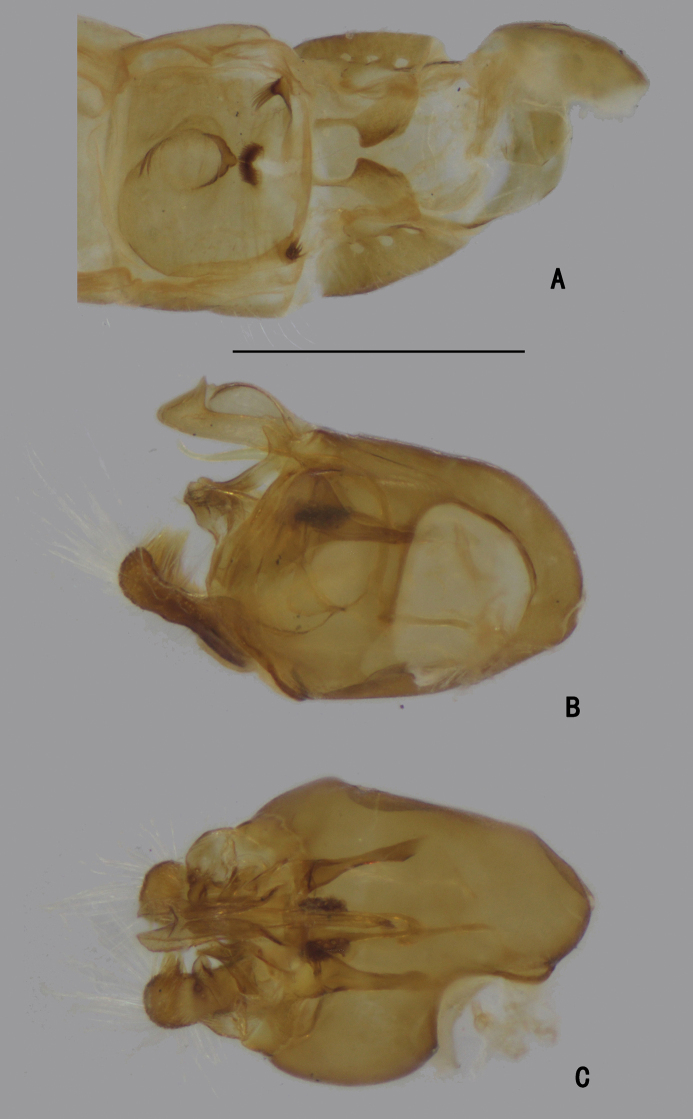
*D.concavus* male **A** abdominal Sternite IV and V, male genitalia removed, ventral view **B** male genitalia, lateral view **C** male genitalia, ventral view. Scale bar: 1 mm.

**Figure 6.**
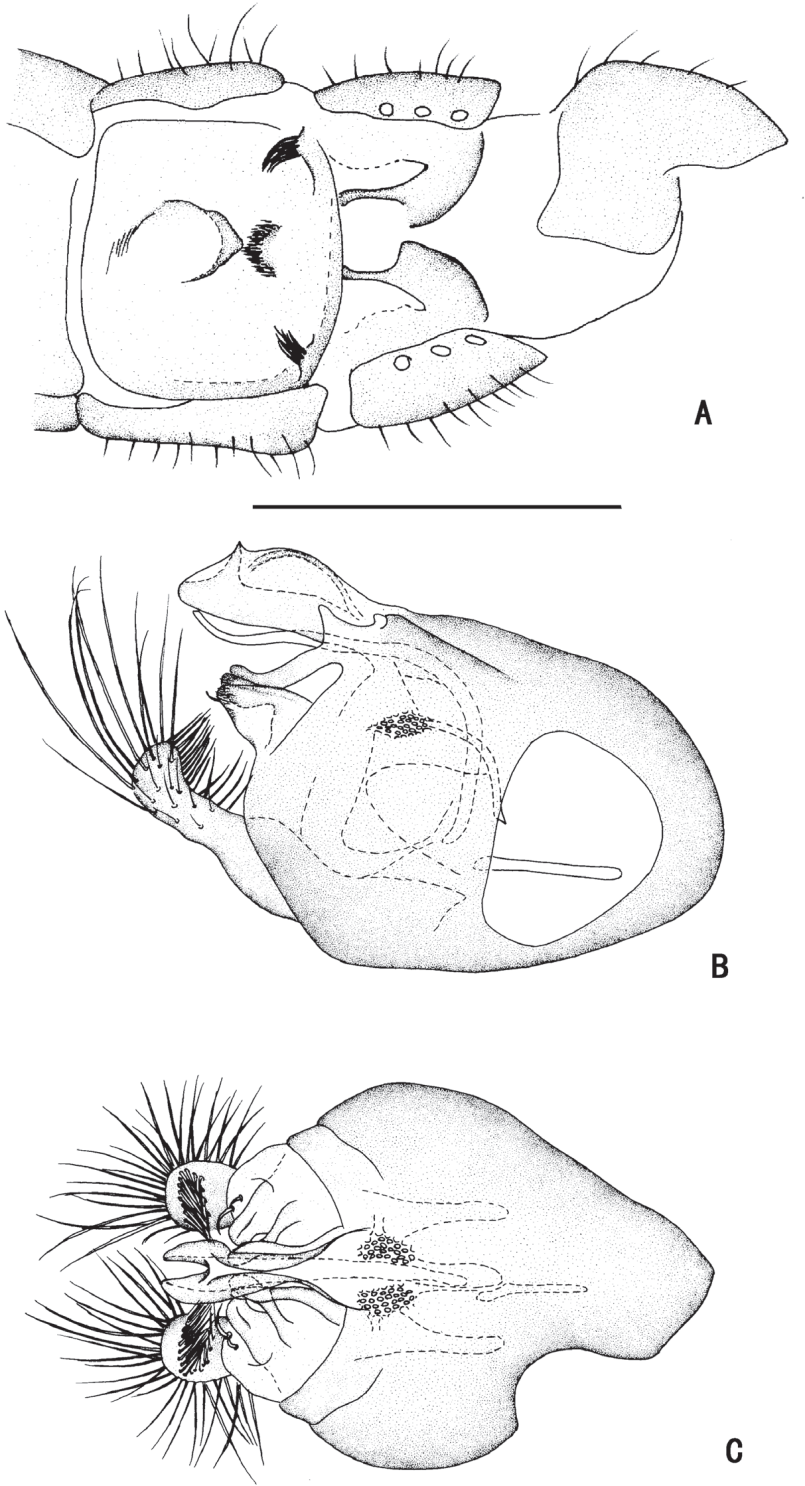
*D.concavus* male **A** abdominal Sternite IV and V, male genitalia removed, ventral view **B** male genitalia, lateral view **C** male genitalia, ventral view. Scale bar: 1 mm.

***Abdomen*** nearly as long as head and thorax combined, dark metallic green with pale gray pollinosity. Abdomen with pale pubescence. Sternite IV medially with an obtuse anterior process and a tubercle bearing bundle of brown bristles, each latero-posterior corner with a tubercle bearing bundle of brown bristles (Figs 5A, 6A). Sternite V split into pair of sclerites, each sclerite ginkgo leaf-like in shape (Figs 5A, 6A).

***Male genitalia*** (Figs 5B, C, 6B, C): Epandrium slightly longer than wide. Epandrial lobe long, wavy, band-like, with an acute basal process, a short bristle at tip. Surstylus thick, lamellated, with two short spines. Hypandrium short thick, apically with a shallow, V-shaped apical incision. Cercus rather short (1/3 as long as epandrium), spoon-shaped, with dark yellow hairs on outer surface, apical one long (nearly as long as cercus), and subapically with group of dense erect dark yellow bristles on inner surface.

**Female**. Unknown.

#### Distribution.

China (Tibet).

#### Remarks.

The new species belongs to the *flexus* subgroup of *D.fenestratus* group. This new species has wing characteristics similar to that of *D.strenus* sp. nov., but the latter can be separated from *D.concavus* by It_3–5_, which is normal and has no ventral suture (MSSC).

#### Etymology.

New species name refers to the concave It_1_ of males.

### ﻿ Diostracus fasciculatus sp. nov.

Taxon classificationAnimaliaDipteraDolichopodidae

24D3C6F5-D235-5E4E-B0EC-EA256EE7F988

https://zoobank.org/49078BA3-AAB9-4799-B295-B557A671788D

**Figure 7.**
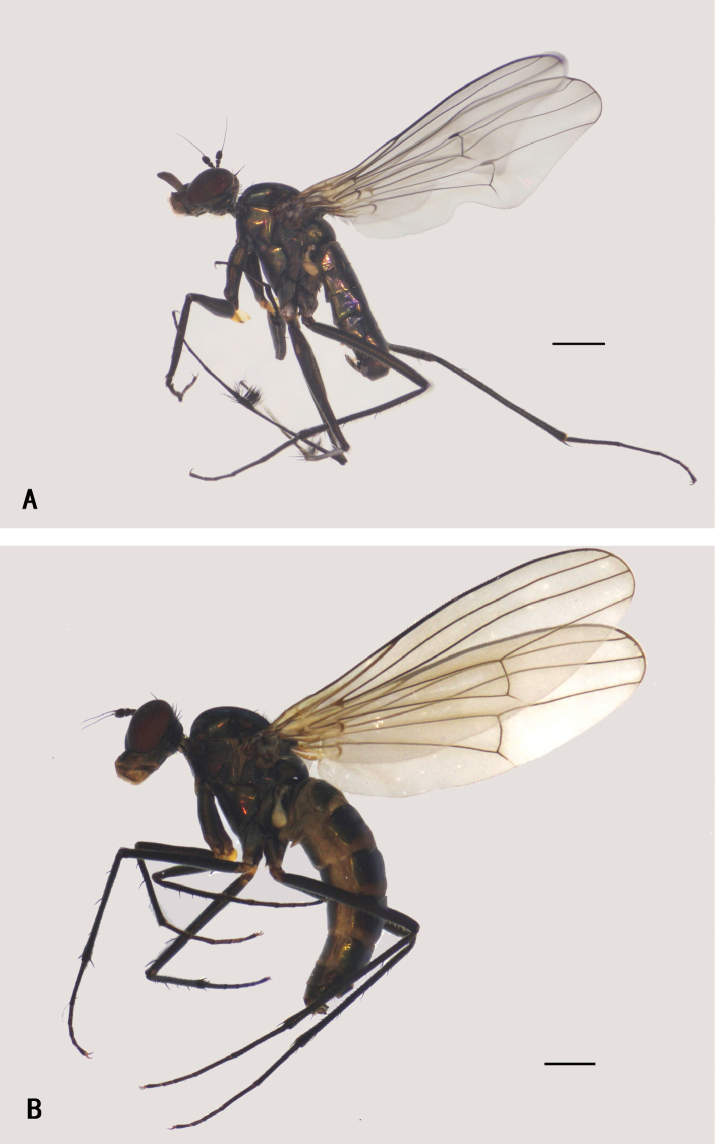
*D.fasciculatus***A** male; lateral view **B** female, lateral view. Scale bars: 1 mm.

**Figure 8.**
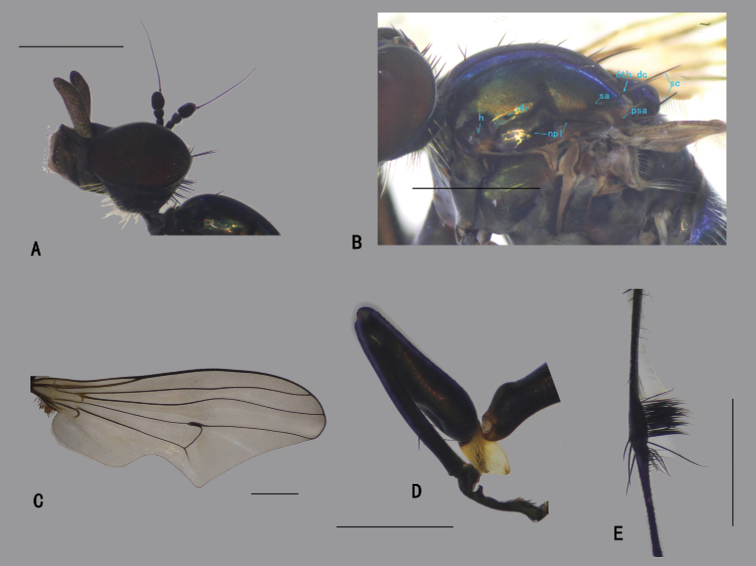
*D.fasciculatus* male **A** head, lateral view **B** thorax, lateral view, show the bristles **C** wing **D**FI, anterior view **E** part of TII and IIt_1_, anterior view. Scale bars: 1 mm.

**Figure 9.**
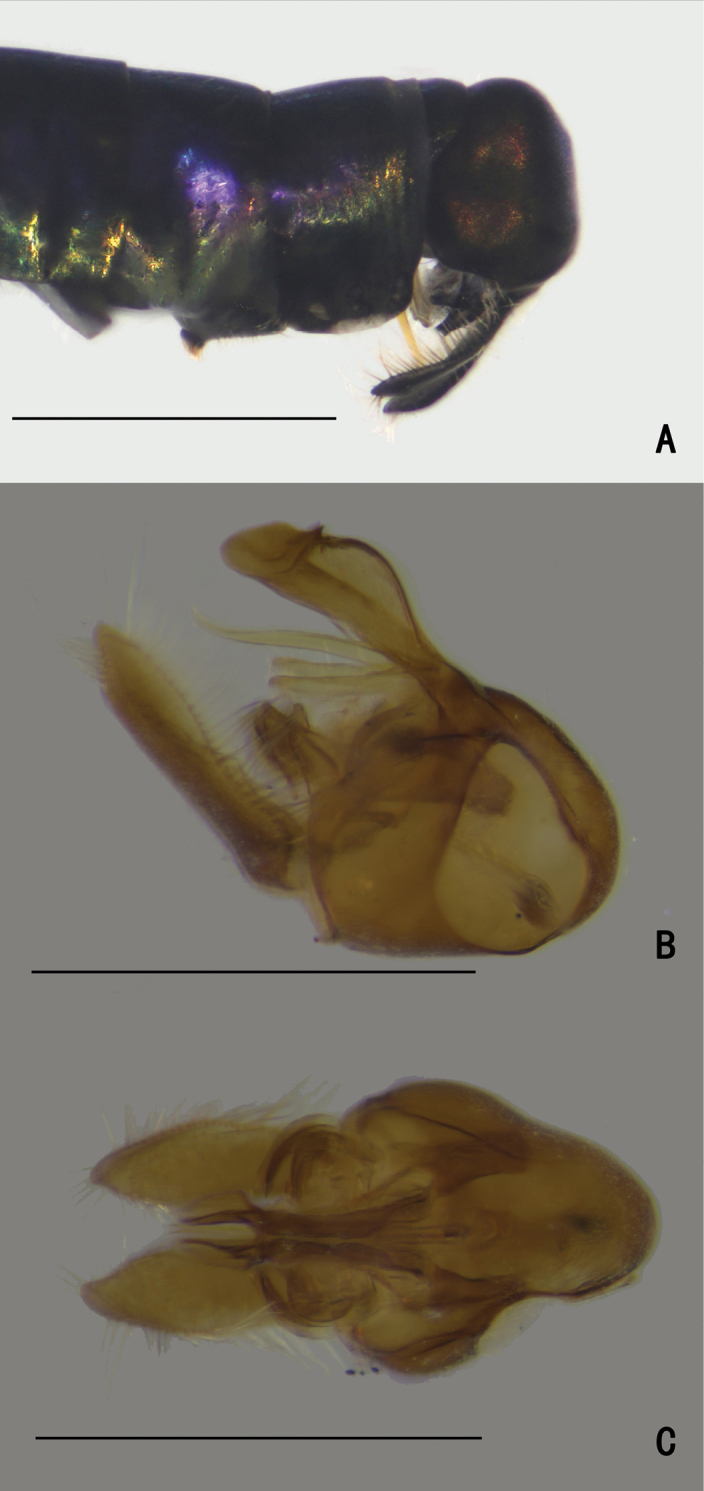
*D.fasciculatus* male **A** abdomen, lateral view **B** male genitalia, lateral view **C** male genitalia, ventral view. Scale bars: 1 mm.

**Figure 10.**
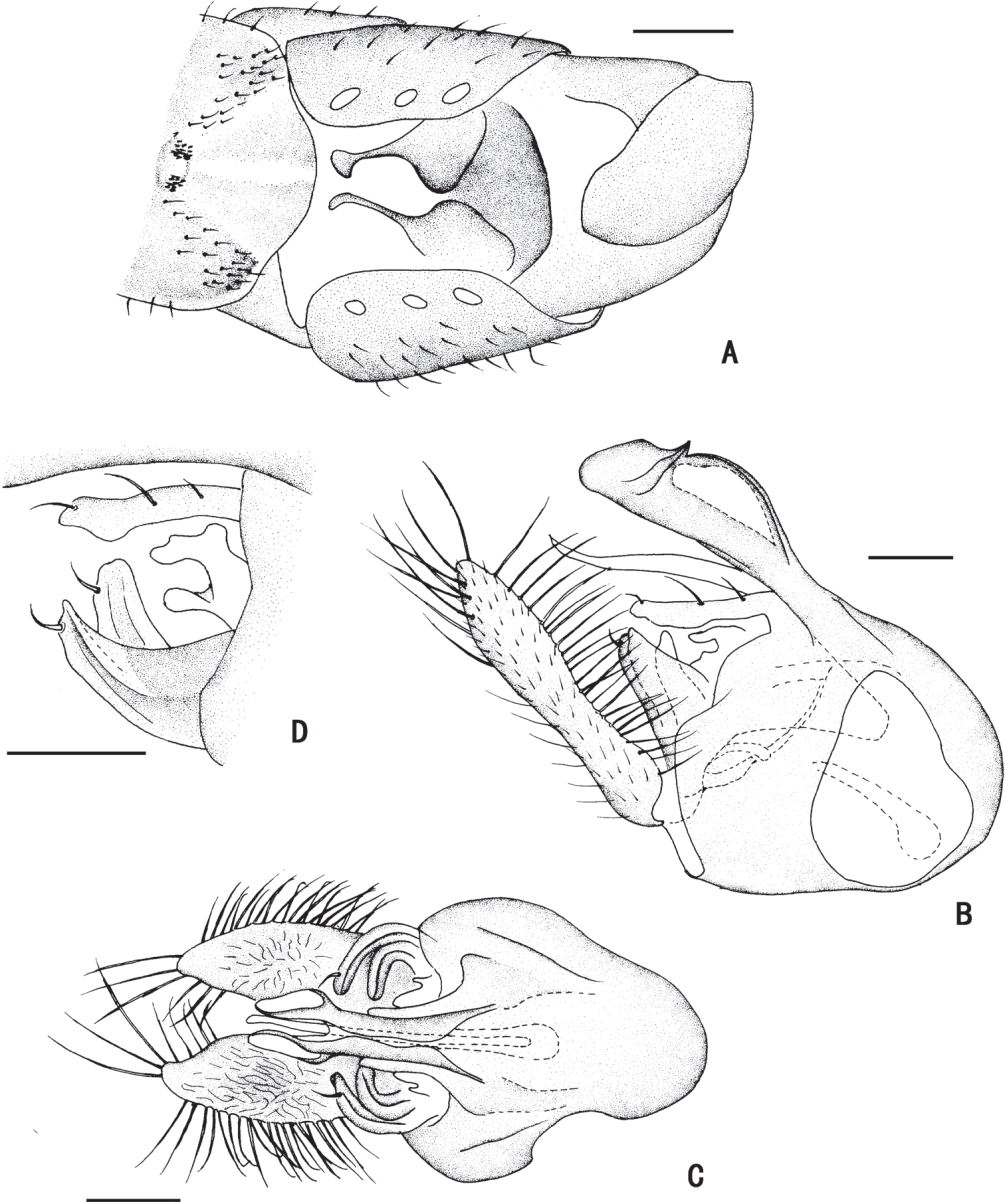
*D.fasciculatus* male **A** abdominal Sternite IV and V, male genitalia removed, ventral view **B** male genitalia, lateral view **C** male genitalia, ventral view **D** surstylus, ventral view. Scale bars: 0.2 mm.

**Figure 11.**
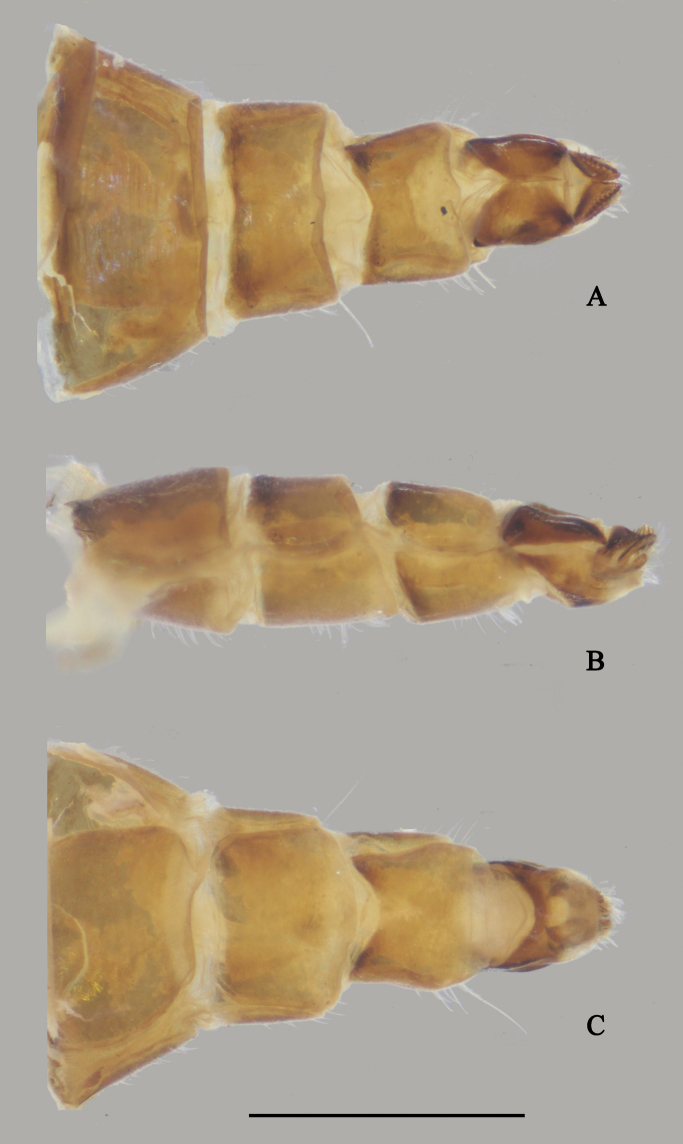
*D.fasciculatus* female, abdomen **A** dorsal view **B** lateral view **C** ventral view. Scale bar: 1 mm.

Figs 7 , 8 , 9 , 10 , 11 , 31C , 32C

#### Type material.

***Holotype***: China • ♂, China: Tibet, Shigatse, Yatung County (27°48'N, 88°90'E), 2700–3200 m, 2018. VII. 13, leg. Yajun Zhu. ***Paratypes***: • 3 ♂♂ 2 ♀♀, same data as for holotype; 11 ♂♂, 4 ♀♀, same data as for preceding, but Pamaimang, 3350 m, 2018. VII. 14.

#### Diagnosis.

MSSC: posterior margin of wing somewhat prolonged along vein CuA_1_. TII and TIII prolonged; apex of TII swollen with two rows of narrow, flat, willow leaf-like ventral bristles, row of long av and pale curve ventral hairs, row of erect pv along whole length, apically with three long bristles; It_1_ swollen at extreme base, with rows of long curved posterior bristles.

#### Description.

**Male** (Fig. 7A). Body length 5.0–5.4 mm; wing length 5.8–6.2 mm.

***Head*** (Fig. 8A) dark metallic green with pale gray pollinosity. Eyes separated; face widened towards clypeus. Hairs and bristles on head black; lower postocular bristles including posteroventral hairs pale. Ocellar tubercle distinct, with pair of strong oc, without posterior hairs; vt rather short, 0.5 × as long as oc, nearly as long as pvt. Antenna black; scape without dorsal bristle; first flagellomere subtriangular, 1.5 × longer than wide; arista apicodorsal, 4.5 × as long as first flagellomere, nearly bare. Proboscis blackish with pale hairs; palpus lobate, 4 × as long as broad, blackish with a purple luster, without distinctive bristle.

***Thorax*** (Fig. 8B) dark metallic green with pale gray pollinosity. Hairs and bristles on thorax black; six mostly hair-like dc except posterior most one dc longest and thick; acr absent; one h, one ph, two npl, one sa, one psa; scutellum with pair of long sc. Propleuron with two or three sparse short pale hairs on upper portion and one or two short pale hairs on lower portion.

***Legs*** nearly entirely black except fore trochanter dark yellow; claws well developed, empodium and pulvilli reduced. Hairs and bristles on legs black except those on coxae pale. CI without distinctive bristle, but with dense erect anterior pale hairs on apical 1/4; CII nearly bare; CIII with blackish bristle at extreme apex. Fore trochanter elongated, with hook-like posterior process (Fig. 8D). FI distinctly thickened (Fig. 8D); TI slightly thickened, weakly curved, with one ad at basal 1/3, two pd (one at apical 1/3 outstanding), row of weak pv along whole length; It_1_ shortened, concave ventrally, anterior ventral margin expanded into a lobe and recurved, with short bristles apically (Fig. 8D); It_2_ with a finger-like lobe at extreme base, corresponding to the cavity of It_1_, apical half thickened with short dense pv. FII somewhat flattened laterally, with three ad on apical half; apex of TII swollen with two rows of narrow flat willow leaf-like ventral bristles, row of long av (anterior ones somewhat curved), rows of pale long ventral hairs (2–3 × longer than TII depth, curved), row of erect pv along whole length (as long as TII depth), apically with three long bristles (Fig. 8E); It_1_ swollen at extreme base, with rows of long curved posterior bristles (Fig. 8E). FIII with two av on apical 1/3, rows of sparse pale ventral hairs along whole length (less than FIII depth); TIII with four ad, three pd, without outstanding ventral bristle, apically with two strong long bristles. Relative lengths of tibia and five tarsomeres: LI 5.3: 0.8: 1.5: 1.8: 0.7: 0.75; LII 7.3: 2.9: 1.4: 0.85: 0.45: 0.75; LIII 8.9: 3.3: 2.3: 1.2: 0.65: 0.85.

***Wing*** (Fig. 8C) hyaline, indistinctly tinged grayish; veins dark brown, R_4+5_ curved at apical 1/3; crossvein m-cu acutely and deeply arched to vein M_1_, forming a hairpin curve, with a slender jet-black brand inside hairpin curve; posterior margin somewhat prolonged along vein CuA_1_. Squama brown with brown hairs. Halter brown.

***Abdomen*** (Fig. 9A) nearly as long as head and thorax combined, dark metallic green with pale gray pollinosity. Abdomen with pale pubescence. Sternite IV medially with pair of obtuse anterior process (very close), bearing bundle of brown hairs (Fig. 10A). Sternite V split into pair of sclerites, each sclerite ginkgo leaf-like (Fig. 10A).

***Male genitalia*** (Figs 9B, C, 10B, C): Epandrium slightly longer than wide. Epandrial lobe pale and lamellated, band-like, elongated, apically with short hairs. Surstylus thick, bent inwards, apically furcated. Hypandrium thick, apically with a deep, U-shaped incision in ventral view. Cercus broad, leaf-like, nearly as long as epandrium, with long dark yellow hairs along margins.

**Female** (Fig. 7B). Body length 4.9–6.7 mm; wing length 6.1–7.9 mm. Nearly as same as male, but: antenna (Fig. 31C) scape with two short dorsal bristles, first flagellomere somewhat prolonged, 1.2 × longer than width, arista apicobasal, 4.6 × longer than first flagellomere. Proboscis yellowish brown with blackish edge; palpus relatively smaller than males, not reaching apex of proboscis. Six weak dc, except posterior most dc longest and thicken. Propleuron with one or two pale curved hairs on lower portion. Legs black, except trochanters yellowish brown. CI without distinctive bristle or hair, but with short pale anterior hairs on lower position; FI without distinct bristles; TI with one ad at basal 1/4, four pd, apically with a bristle and comb of anterior bristles; FII with two anterior bristles on apical 1/3; TII with three ad, two pd, apically with five bristles; FIII with three anterior bristles on apical 1/3; TIII with four ad, two pd, 4–6 weak v, apically with two bristles. Wing (Fig. 32C): m-cu straight, forming acute angle with CuA_1_. Halter yellow.

***Female terminalia*** (Fig. 11): Abdominal tergite VIII divided into two sclerites; epiproct split into pair of triangular hemitergites, apically with row of nine strong curved spines; dorsal lobes of cercus triangulated, with dark yellow bristles; ventral lobes of cercus short, apex rounded, with long yellow hairs.

#### Distribution.

China (Tibet).

#### Remarks.

The new species belongs to the *flex* subgroup *D.fenestratus* group. The species is unique for the shape of wings and It_1–2_, and the prolonged TII and TIII which have relatively short IIt_1_ and IIIt_1_. Females are characterized by an arched crossvein m-cu, and the crossvein vertical adjunct to vein CuA_1_; the trochanters are black.

#### Etymology.

The new species name refers to the cluster of bristles on apex of TII of males.

### ﻿ Diostracus laetus sp. nov.

Taxon classificationAnimaliaDipteraDolichopodidae

D788255C-399C-51BB-A28A-C817D1BE5E4C

https://zoobank.org/43CC31E5-3F0A-4277-8734-A41164D50B41

**Figure 12.**
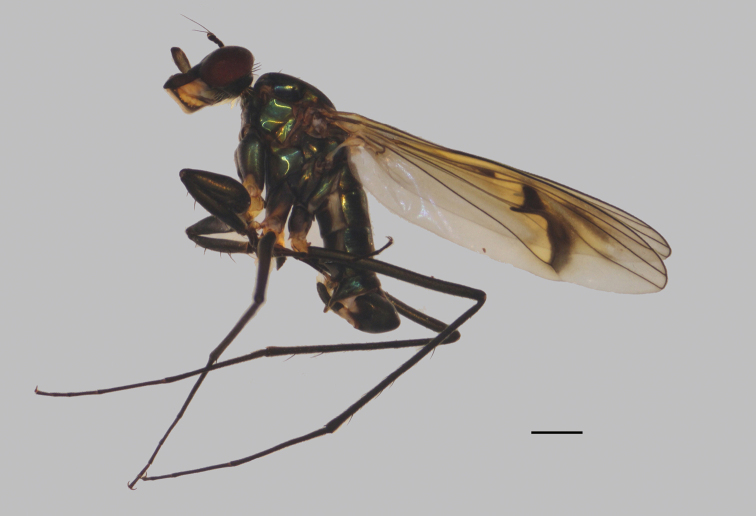
*D.laetus*, male, lateral view. Scale bar: 1 mm.

**Figure 13.**
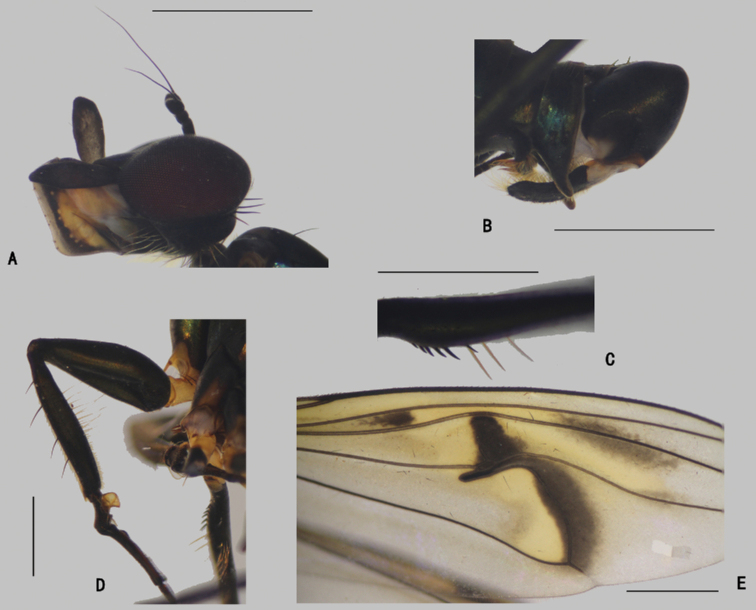
*D.laetus* male **A** head, lateral view **B** abdomen, lateral view **C** base of FII, anterior view **D**LI, anterior view **E** part of wing. Scale bars: 1 mm.

**Figure 14.**
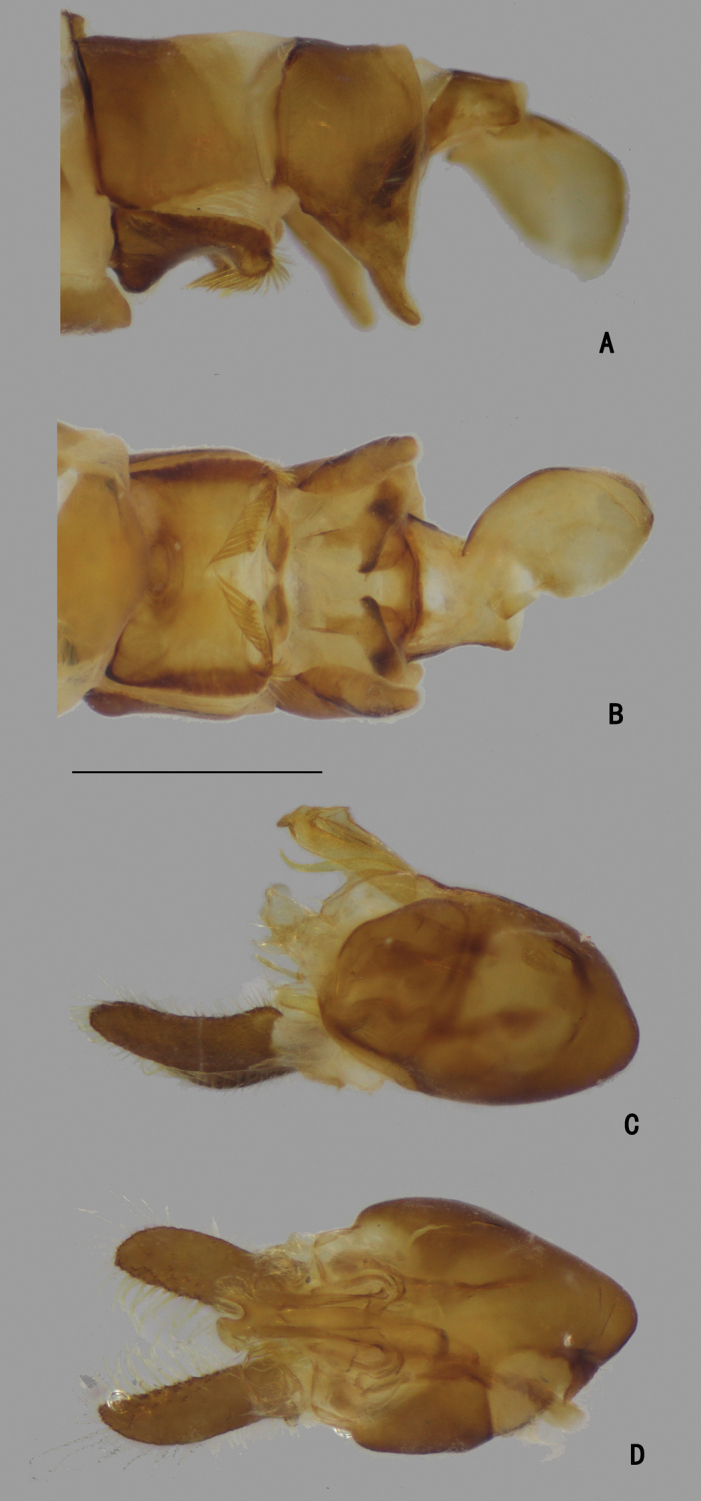
*D.laetus* male **A** abdominal Sternite IV and V, male genitalia removed, lateral view **B** abdomen, male genitalia removed, ventral view **C** male genitalia, lateral view **D** male genitalia, ventral view. Scale bar: 1 mm.

**Figure 15.**
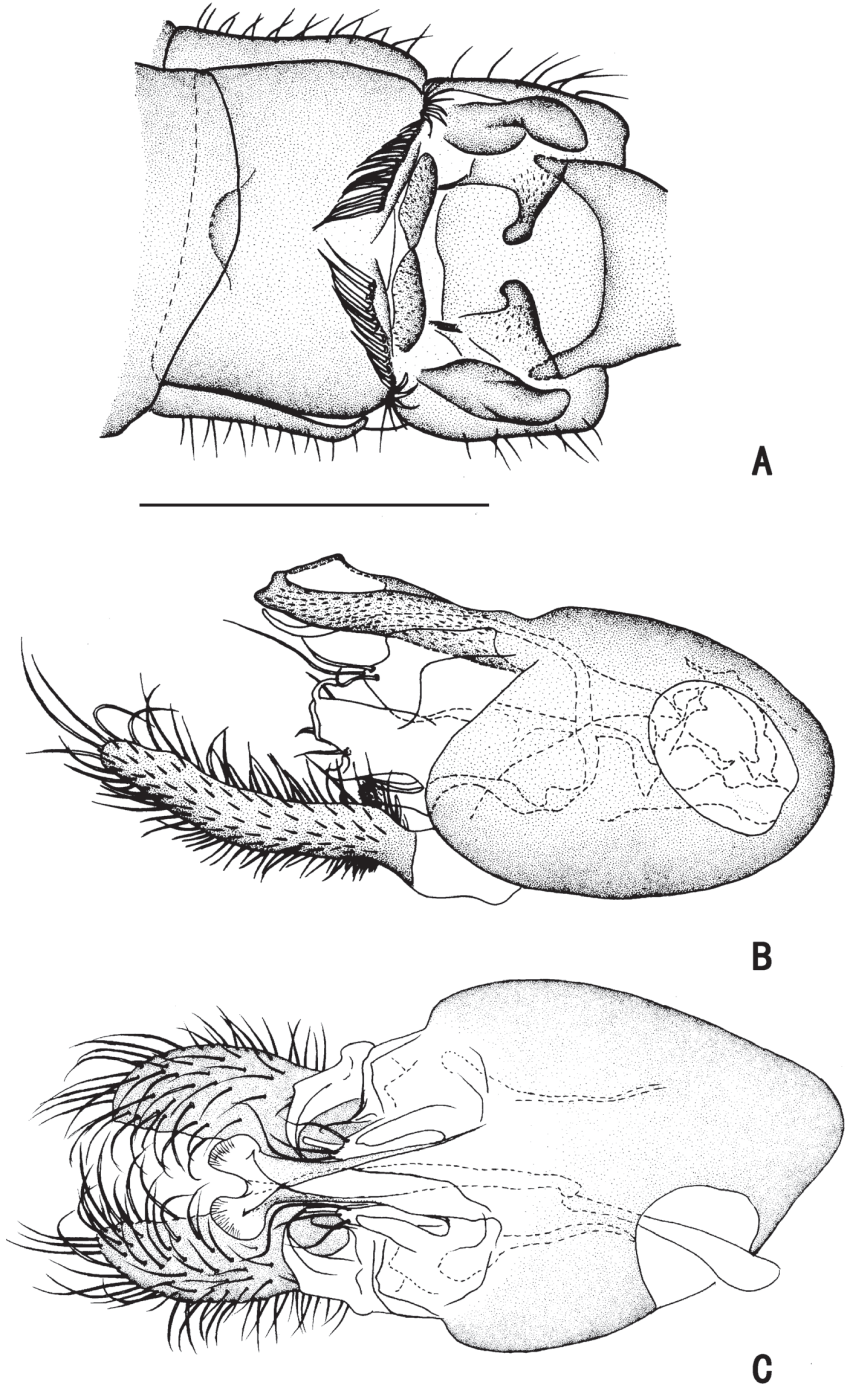
*D.laetus* male **A** abdominal Sternite IV and V, male genitalia removed, ventral view **B** male genitalia, lateral view **C** male genitalia, ventral view. Scale bar: 1 mm.

Figs 12 , 13 , 14 , 15

#### Type material.

***Holotype***: China • ♂, China: Tibet, Shigatse, Yatung County (27°48'N, 88°90'E), 2700–3200 m, 2018. VII. 13, leg. Yajun Zhu.

#### Diagnosis.

MSSC: Wings with dark and yellow markings at middle. CI with a brown curved anterior spine at extreme apex; CII with row of four brown anterior spines along apical edge; fore trochanter with row of upwards curved bristles along basal edge. FI with short upwards curved ventral bristles on basal 1/4; TI thickened, with rows of pale ventral hairs on apical 3/4, and apically with two long wavy posterior bristles; FII with row of long av and posteroventral hairs.

#### Description.

**Male** (Fig. 12). Body length 7.4 mm; wing length 8.2 mm.

***Head*** (Fig. 13A) dark metallic green with pale brown pollinosity. Eyes separated; face widened towards clypeus. Hairs and bristles on head black; lower postocular bristles including posteroventral hairs pale. Ocellar tubercle distinct, with pair of oc (broken), pair of posterior hairs; vt weak, slightly shorter than pvt. Antenna black; scape without dorsal bristle; first flagellomere subrectangular, 1.3 × longer than wide; arista apicodorsal, 5.3 × as long as first flagellomere, nearly bare. Proboscis yellowish brown with blackish edge; palpus lobate, 2.3 × as long as broad, blackish with a purple luster, without distinctive bristle.

***Thorax*** dark metallic green with pale brown pollinosity. Hairs and bristles on thorax black; six mostly hair-like dc except posterior most one dc longest and thick; acr absent; one h, one ph, two npl, one sa, one psa; scutellum with pair of long sc. Propleuron with two or three sparse short pale curved hairs on upper portion and two long pale curved hairs on lower portion.

***Legs*** nearly entirely black except extreme apexes of coxae and trochanters brownish yellow; claws well developed, empodium and pulvilli reduced. Hairs and bristles on legs black. CI with short sparse pale hairs on anterior surface, and a brown curved anterior spine at extreme apex (Fig. 13D); CII with row of four brown anterior spines along apical edge; CIII nearly bare. Fore trochanter with ridge and row of upwardly curved bristles along basal edge (Fig. 13D). FI distinctly thickened, with short upwards curved ventral bristles on basal 1/4; TI thickened, with four ad, one pd at middle, rows of pale ventral hairs on apical 3/4, and apically with two long wavy posterior bristles (Fig. 13D); It_1_ shortened, apex expanded, concave ventrally, anterior ventral margin expanded into a lobate, with comb of bristles on anterior surface (Fig. 13D); It_2_ with a finger-like lobe at extreme base, with a posteroventral ridge on basal half (Fig. 13D). FII thickened, with row of seven short av spines, row of three long anteroventral hairs and row of long posteroventral hairs (as long as FII depth, somewhat curved on basal 2/3) (Fig. 13C); TII with two ad, apically with two long bristles. FIII without conspicuous hairs and bristles; TIII with three ad, two pd, without outstanding ventral bristle. Relative lengths of tibia and five tarsomeres: LI 5.5: 1.2: 3.0: 3.3: 1.8: 1.0; LII 10.2: 6.4: 3.2: 1.8: 0.9: 1.0; LIII 11.8: 6.2: 4.2: 1.8: 0.9: 1.0.

***Wing*** (Fig. 13E) hyaline, anterior half and area around crossvein m-cu yellowish, with dark cloud on middle of cell R_2+3_, and a stripe of dark cloud along M_1_ and expanding along posterodistal corner of distal cell, dark cloud in subapical portion of distal cell prominent; veins dark brown, R_4+5_ curved at middle, M_1_ curved at apical 1/3; crossvein m-cu acutely and deeply arched to vein M_1_, forming a hairpin curve, with a slender jet-black brand inside hairpin curve, accessory cellula 1.3 × longer than width. Squama brown with brown hairs. Halter yellow with blackish apex.

***Abdomen*** (Fig. 13B) nearly as long as head and thorax combined, dark metallic green with pale brown pollinosity, except edge of sternites, apex of epandrium and base of cercus pale. Abdomen with short sparse pale pubescence. Posterior edge of sternite IV forwards recurved with row of short curve spines (Figs 14A, B, 15A). Lateroposterior corner of tergite V elongated into a triangular process. Sternite V split into pair of sclerites, each sclerite ginkgo leaf-like (Figs 14A, B, 15A).

***Male genitalia*** (Figs 14C, D, 15B, C): Epandrium slightly longer than wide. Epandrial lobe and surstylus pale and lamellated. Epandrial lobe short, band-like, apically with one long and one short bristles. Surstylus irregular in shape, with irregular processes and bristles. Process of subepandrial sclerite exceeding epandrium margin, with short fine pubescence. Hypandrium thick, apically with a shallow, U-shaped incision. Cercus band-like, somewhat bent, with long dark yellow hairs around cercus.

**Female.** Unknown.

#### Distribution.

China (Tibet).

#### Remarks.

The new species belongs to *pulchripennis* subgroup and is quite similar to *D.emotoi*. Both species have same chaetotaxy on FII and they are similar in wing style. However, the new species has no posterior bristles on FI, long ventral hairs and two wavy bristles on TI, and relatively smaller wing accessory cellula (2.5 × as long as wide).

#### Etymology.

The name of new species refers to the bright coloration of male wings.

### ﻿ Diostracus polytrichus sp. nov.

Taxon classificationAnimaliaDipteraDolichopodidae

D806573B-75C5-50BB-9834-04B475CE308D

https://zoobank.org/4318BDD9-0AE4-4A15-96DE-549267680311

**Figure 16.**
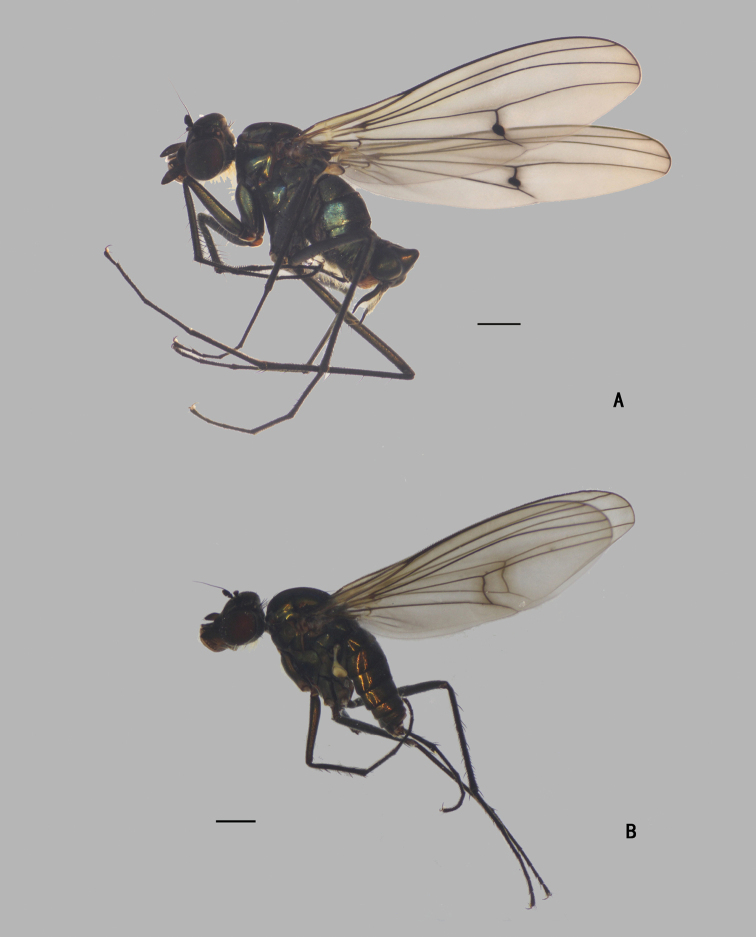
*D.polytrichus***A** male; lateral view **B** female, lateral view. Scale bars: 1 mm.

**Figure 17.**
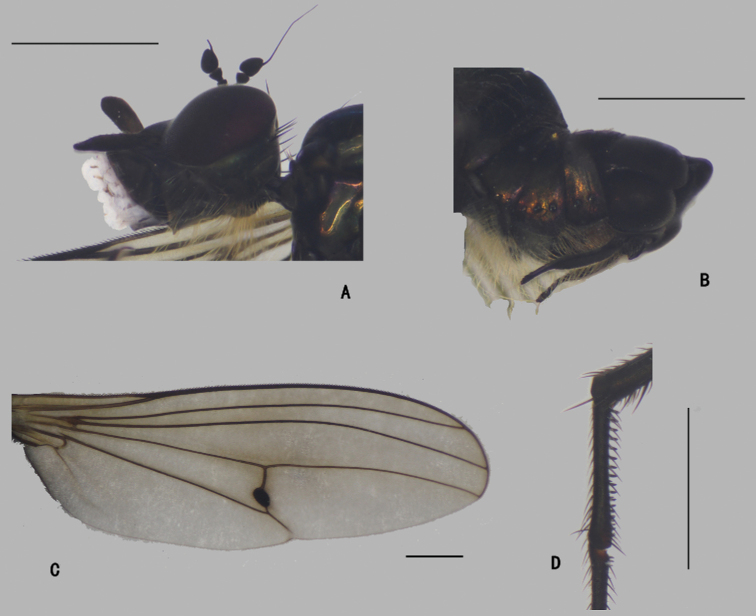
*D.polytrichus* male **A** head, lateral view **B** abdomen, lateral view **C** wing **D**It_1_, anterior view. Scale bars: 1 mm.

**Figure 18.**
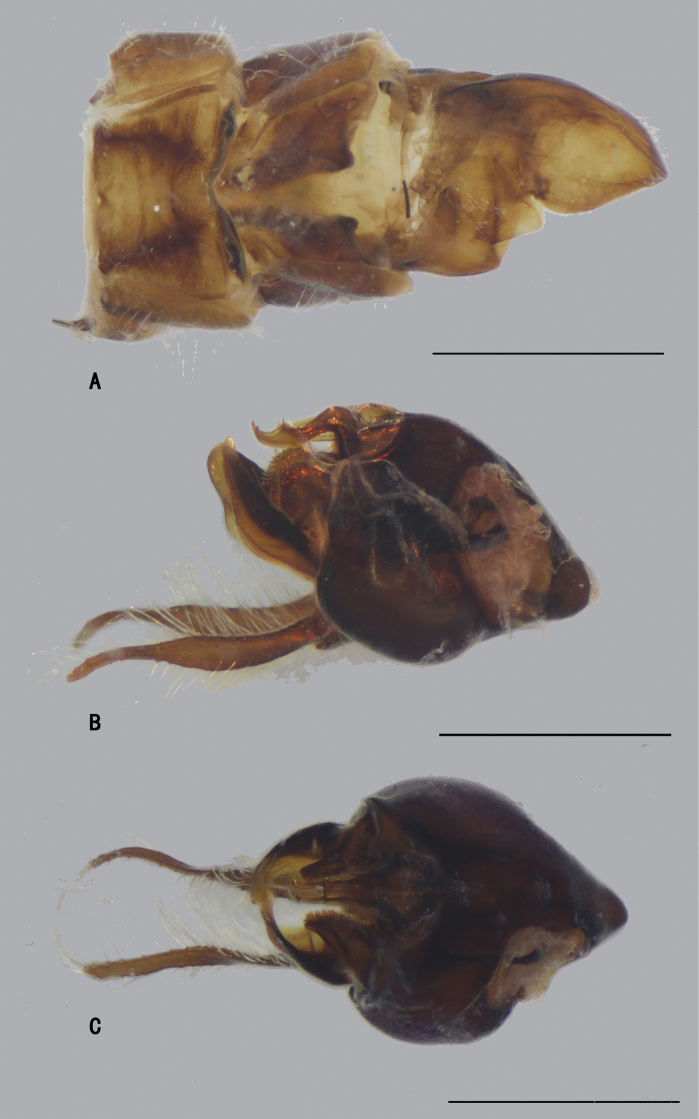
*D.polytrichus* male **A** abdominal Sternite IV and V, male genitalia removed, ventral view **B** male genitalia, lateral view **C** male genitalia, ventral view. Scale bars: 1 mm.

**Figure 19.**
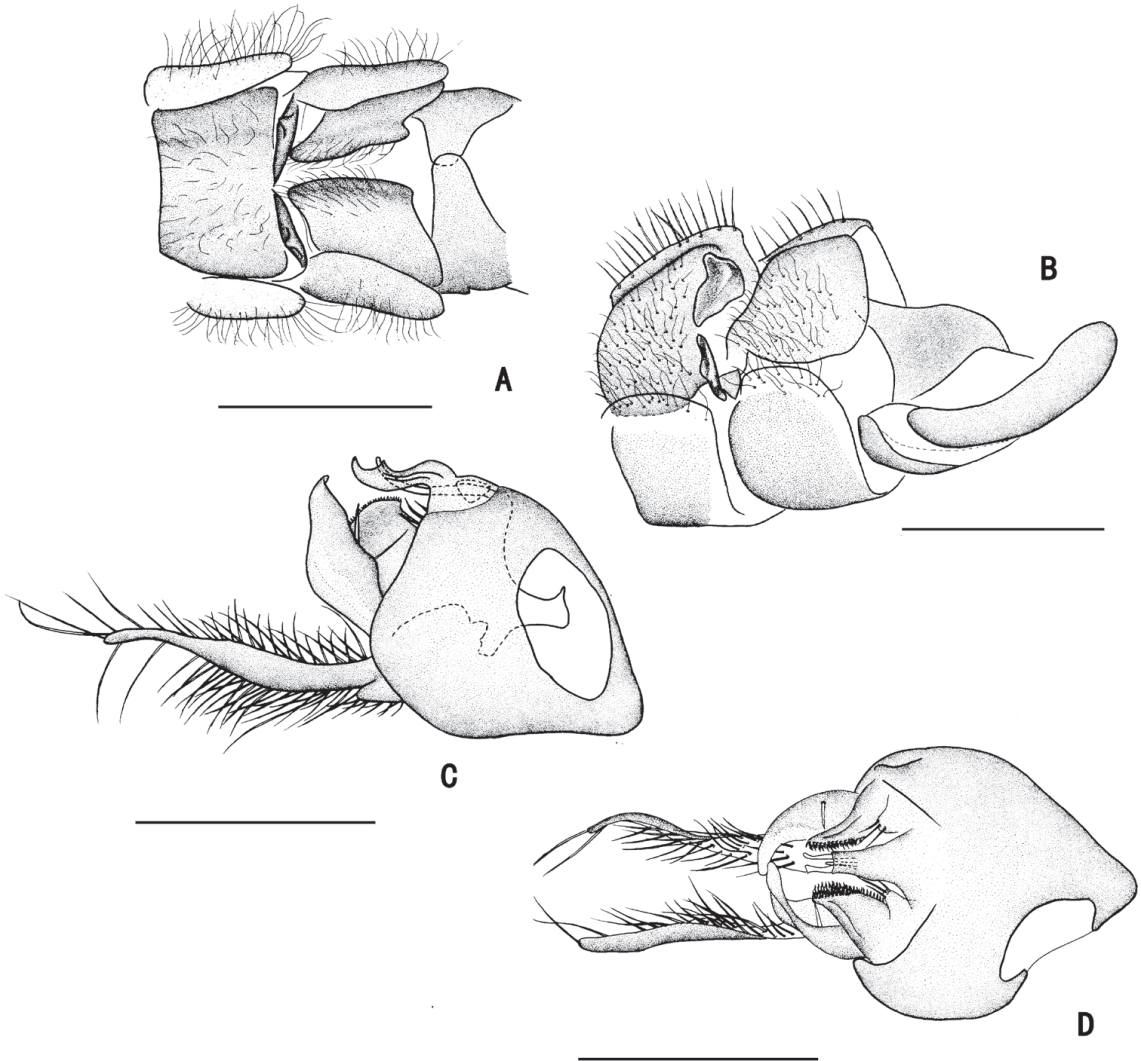
*D.polytrichus* male **A** abdominal Sternite IV and V, male genitalia removed, ventral view **B** abdominal Sternite IV and V, male genitalia removed, lateral view with some angles **C** male genitalia, lateral view **D** male genitalia, ventral view. Scale bars: 1 mm.

**Figure 20.**
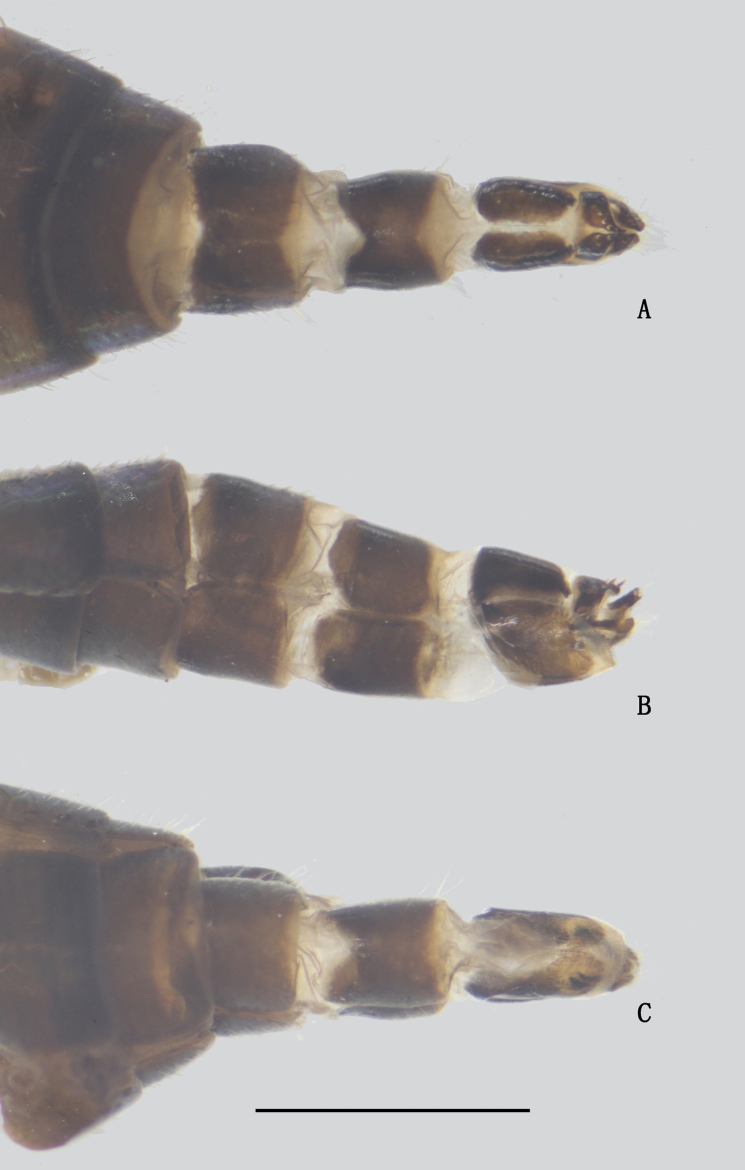
*D.polytrichus* female, abdomen **A** dorsal view **B** lateral view **C** ventral view. Scale bar: 1 mm.

Figs 16 , 17 , 18 , 19 , 20 , 31D , 32E

#### Type material.

***Holotype***: China • ♂, China: Tibet, Shigatse, Yatung County (27°48'N, 88°90'E), Pamaimang, 3350 m, 2018. VII. 14, leg. Yajun Zhu. ***Paratypes***: • 2 ♂♂ 2 ♀♀, same data as for holotype.

#### Diagnosis.

MSSC: palpus black; wing crossvein m-cu nearly straight, with jet-black nodule; halter yellow, with blackish apex. Abdomen with dense ventral hairs.

#### Description.

**Male** (Fig. 16A). Body length 6.6–7.0 mm; wing length 9.4–9.8 mm.

***Head*** (Fig. 17A) dark metallic green with pale gray pollinosity. Eyes separated; face widened towards clypeus. Hairs and bristles on head black; lower postocular bristles including posteroventral hairs brownish. Ocellar tubercle distinct, with pair of strong oc, without posterior hairs; vt rather short, 0.3 × as long as oc, sometimes hair-like; pvt slight shorter than oc. Antenna black; scape bare, or with three or four dorsal bristles; first flagellomere semicircular to subtriangular, 1.2–1.6 × longer than wide; arista apico-dorsal, 4.2–7.5 × as long as first flagellomere, nearly bare. Proboscis blackish with pale hairs; palpus lobate, 2.2–2.5 × as long as broad, blackish with a purple luster, without distinctive bristle.

***Thorax*** dark metallic green with pale gray pollinosity; mesoscutum with two dark brown longitudinal stripes. Hairs and bristles on thorax black; six weak dc except posterior most one dc longest and thick, occasionally with excess dc; acr absent; one weak and one strong h, one ph, two npl, one sa, one psa; scutellum with pair of long sc. Propleuron with one or two sparse long pale hairs on upper portion and group of long pale hairs on lower portion.

***Legs*** nearly entirely black except fore trochanter dark brown; claws well developed, empodium and pulvilli reduced. Hairs and bristles on legs black except those on coxae pale. CI with group of pale curved anterior hairs on basal 1/3, upper ones long, and cluster of erect bristles on apical 1/4; CII nearly bare; CIII with blackish bristle at extreme apex. FI thickened, with two rows of ventral hairs (as long as FI depth), basal ones pale, and one posterior bristle at extreme base; TI with two pd, three d, row of six or seven long pv on apical half, apically with two bristles and comb of anterior bristles; It_1–2_ with rows of pd and pv, ventral surface with short dense fine hairs, It_1_ with row of av spines, extending to It_2_ (Fig. 17D); FII with two rows of av on basal half, one distinct v at middle, row of short av on apical 1/3, row of pale long pv on basal 2/3 (longest ones 2.5 × longer than FII depth); TII with two ad, two pd, apically with four bristles. FIII with four av on middle 1/3, apically with one av; TIII with four ad, four pd, four short ventral bristles, apically with three long bristles and comb of short anterior bristles. Relative lengths of tibia and five tarsomeres: LI 6.9: 3.1: 3.2: 1.7: 1.1: 1.0; LII 5.8: 3.2: 1.4: 0.9: 0.5: 0.8; LIII 7.8: 3.4: 2.5: 1.5: 0.8: 0.9.

***Wing*** (Fig. 17C) hyaline, indistinctly tinged grayish; veins dark brown; crossvein m-cu nearly straight, curved around nodule, with a jet-black nodule. Squama brown with brown hairs. Halter yellow with blackish apex.

***Abdomen*** (Fig. 17B) nearly as long as thorax, dark metallic green with pale gray pollinosity, bent upwards medially. Abdomen with dense long pale pubescence. Sternite V split into pair of sclerites (Figs 18A, 19A, B).

***Male genitalia*** (Figs 18B, C, 19C, D): Epandrium swollen, slightly longer than wide. Epandrial lobe tiny and pale, lamellate, apically with two pale bristles. Surstylus bifurcated, dorsal lobe broad and lamellated, inner surface with dense dentiform bristles, ventral lobe elongated, broad and leaf-like, apical margin pale, with one long inner bristle at middle. Hypandrium relatively small, apically with a deep U-shaped incision in ventral view. Cercus band-like, apical 1/3 becoming narrower, with long yellow hairs along margins.

**Female** (Fig. 16B). Body length 5.4–6.0 mm; wing length 6.8–7.9 mm. Nearly as same as male, but: ocellar tubercle without posterior hairs, antenna (Fig. 31D) scape with two short dorsal bristles, first flagellomere semicircular, nearly as long as width, arista apicobasal, 5.6 × longer than first flagellomere. Proboscis blackish; palpus relatively smaller than males, not reaching apex of proboscis. Seven weak dc, except posterior most dc longest and thicken. Propleuron with two or three sparse short pale curved hairs on upper portion and group of five or six sparse long pale curved hairs on lower portion. Legs black. CI with erect pale hairs on anterior surface, ones on lower portion black and thick; FI with one preapical pv; TI with four pd, two pv, apically with three bristles and comb of short anterior bristles; FII with one strong preapical av and one weak preapical pv; TII with three ad, two pd, apically with four strong bristles; FIII with one strong preapical av and one weak preapical pv; TIII with three ad, apically with three bristles. Wing (Fig. 32E): m-cu straight, forming obtuse angle with CuA_1_; area around m-cu tingled with blackish ash. Halter yellow with blackish apex.

***Female terminalia*** (Fig. 20): Abdominal segments VII and VIII slender; tergite VIII divided into two sclerites; epiproct split into pair of hemitergites, apically with row of five strong curved spines; dorsal lobes of cercus finger-like, somewhat elongated, with dark yellow bristles; ventral lobes of cercus short, apex rounded.

#### Distribution.

China (Tibet).

#### Remarks.

The new species is quite similar to *D.tibetensis*, but the cerci of new species are lamellate with broad base. Females of the new species are characterized by the semicircular first flagellomere of antenna, the straight crossvein m-cu, and the blackish apex of halter.

#### Etymology.

The name of the new species refers to the dense abdominal ventral hairs.

### ﻿ Diostracus strenus sp. nov.

Taxon classificationAnimaliaDipteraDolichopodidae

CEEE1408-BE8D-5244-865C-4D1D57F82387

https://zoobank.org/DD9B81DF-05D1-485A-81E8-572D667BA73F

**Figure 21.**
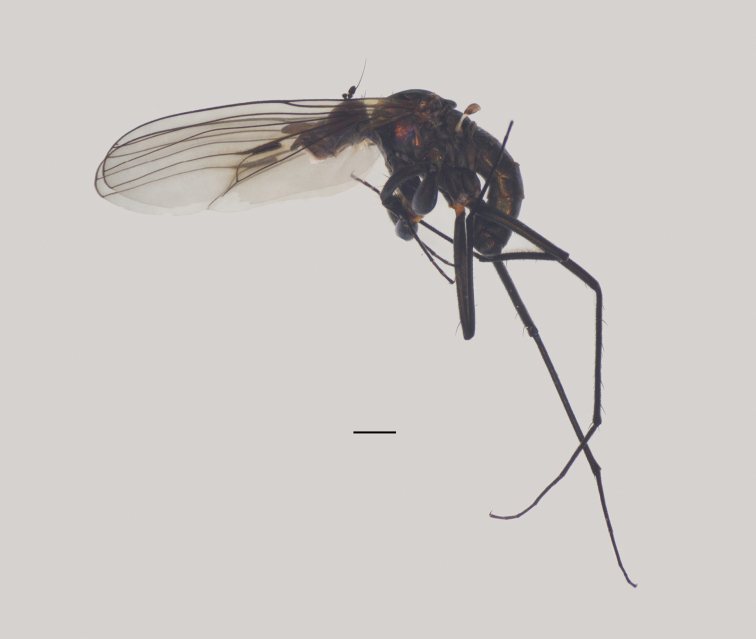
*D.strenus*, male; lateral view. Scale bar: 1 mm.

**Figure 22.**
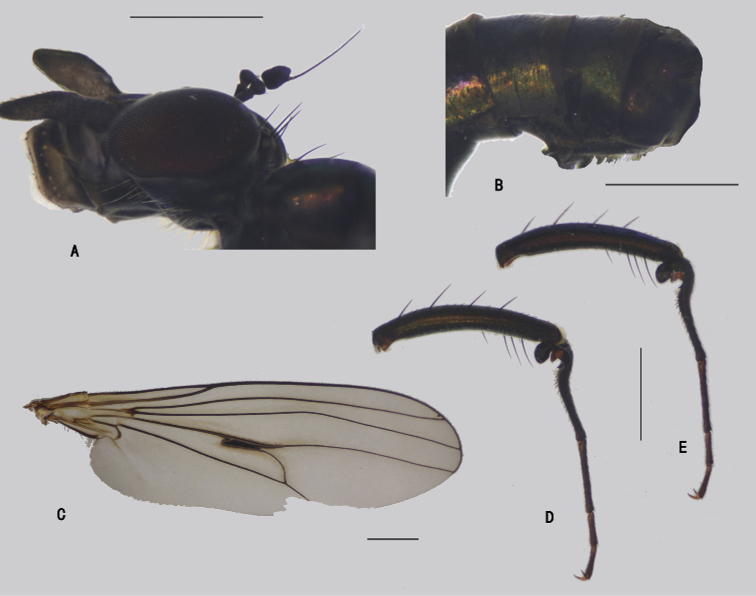
*D.strenus* male **A** head, lateral view **B** abdomen, lateral view **C** wing **D**TI and It, anterior view **E**TI and It, posterior view. Scale bars: 1 mm.

**Figure 23.**
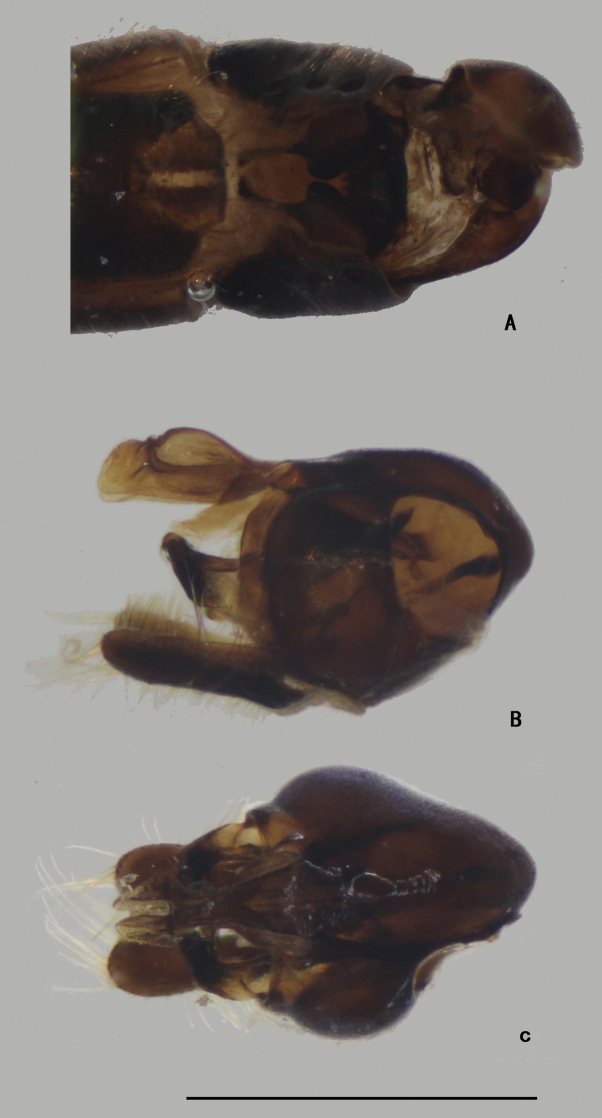
*D.strenus* male **A** abdominal Sternite IV and V, male genitalia removed, ventral view **B** male genitalia, lateral view **C** male genitalia, ventral view. Scale bar: 1 mm.

**Figure 24.**
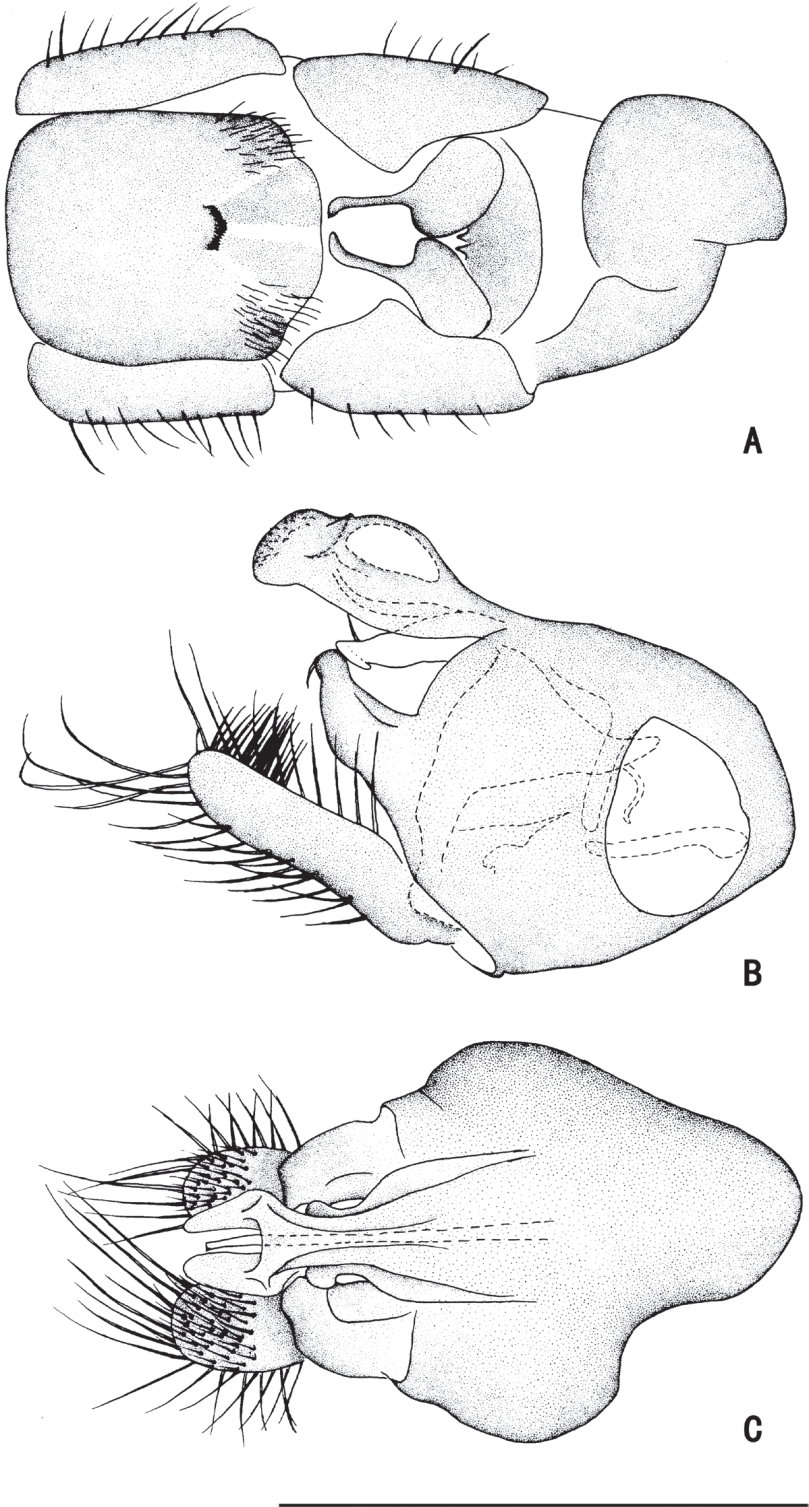
*D.strenus* male **A** abdominal Sternite IV and V, male genitalia removed, ventral view **B** male genitalia, lateral view **C** male genitalia, ventral view. Scale bar: 1 mm.

Figs 21 , 22 , 23 , 24

#### Type material.

***Holotype***: China • ♂, Tibet, Shigatse, Yatung County (27°48'N, 88°90'E), 2700–3200 m, 2018. VII. 13, leg. Yajun Zhu. ***Paratype***: • 1 ♂, same data as for holotype but Pamaimang, 3350 m, 2018. VII. 14.

#### Diagnosis.

MSSC: dark and robust fly; FI and TI distinctly thickened; posterior ventral margin of It_1_ and anterior ventral margin of It_2_ expanded into auriform lobes; crossvein m-cu acutely and deeply arched to vein M_1_, forming a ‘h’-shaped curve, with a jet-black mark inside curve.

#### Description.

**Male** (Fig. 21). Body length 6.0 mm; wing length 8.0 mm.

***Head*** (Fig. 22A) dark metallic green with pale brown pollinosity. Eyes separated; face widened towards clypeus. Hairs and bristles on head black; lower postocular bristles including posteroventral hairs pale. Ocellar tubercle distinct, with pair of strong oc, without posterior hairs; vt short, 0.7 × as long as oc, nearly as long as pvt. Antenna black; scape with a weak dorsal bristle at basal 1/3; first flagellomere subtriangular, 1.5 × longer than wide; arista apicodorsal, 3 × as long as first flagellomere, nearly bare. Proboscis blackish with pale hairs; palpus lobate, 3.5 × as long as broad, blackish with a purple luster, without distinctive bristle.

***Thorax*** dark metallic green with pale brown pollinosity. Hairs and bristles on thorax black; six mostly hair-like dc except 1^st^ and 6^th^dc long and thick; acr absent; one h, one ph, two npl, one sa, one psa; scutellum with pair of sc. Propleuron with two or three sparse, short, pale hairs on upper portion and one or two short pale hairs on lower portion.

***Legs*** nearly entirely black except fore and mid trochanters dark yellow; claws well developed, empodium and pulvilli reduced. Hairs and bristles on legs black except those on coxae pale. CI without distinctive bristle, but with dense, erect, pale, anterior hairs on apical 1/4; CII with cluster of black bristles at extreme apex; CIII nearly bare. Fore trochanter elongated, with lobate posterior process. FI distinctly thickened, with group of pale hairs on apical 1/5 (less than FI depth) (Fig. 22D, E); TI distinctly thickened, curved, with three ad on basal half, two pd on apical 1/3, row of five long pv on apical 1/3, apically with comb of short pale av spines (Fig. 22D, E); It_1_ shortened, concave ventrally, posterior ventral margin expanded into a auriform lobe (Fig. 22D, E); It_2_ thickened and recurved, somewhat flattened dorsoventrally, with anterior ventral margin at extreme base expanded into an auriform lobe, corresponding to the auriform lobe of It_1_, a spine-like lobe at basal 1/4, and two rows of short pv spines (Fig. 22D, E). FII with rows of pale postoventral hairs (as long as FII depth), and one ad at apical 1/8; TII with rows of curved ventral hairs on basal 2/3 (longest ones 2 × longer than TII depth), three weak ad, two weak pd, apically with two long bristles. FIII with two ad on apical 1/6, rows of sparse pale ventral hairs on basal half (less than FIII depth); TIII with five ad, three pd, without outstanding ventral bristle, apically with two bristles. Relative lengths of tibia and five tarsomeres: LI 5.7: 0.6: 2.8: 2.1: 1.0: 1.0; LII 8.8: 5.2: 2.2: 1.3: 0.8: 1.0; LIII 10.3: 4.7: 3.0: 1.4: 0.7: 1.0.

***Wing*** (Fig. 22C) hyaline, indistinctly tinged grayish; veins dark brown; crossvein m-cu acutely and deeply arched to vein M_1_, forming a ‘h’-shaped curve, with a jet-black mark inside curve. Squama brown with brown hairs. Haltere blackish with pale knob.

***Abdomen*** (Fig. 22B) nearly as long as head and thorax combined, dark metallic green with pale gray pollinosity. Abdomen with pale pubescence. Sternite IV medially with an obtuse process, and one tubercle bearing bundle of brown bristles, latero-posterior corner with brown bristles (Figs 23A, 24A). Sternite V split into pair of sclerites, each sclerite ginkgo leaf-like (Figs 23A, 24A).

***Male genitalia*** (Figs 23B, C, 24B, C): Epandrium slightly longer than wide. Epandrial lobe elongated, lamellated, apically with two short bristles. Surstylus thick, apex dark, bending inwards, with a finger-like inner process. Hypandrium thick, apically with a shallow, V-shaped incision. Cercus straight and clavated, inner surface somewhat hollow, with long pale hairs along margin, apical half with dense, erect, dark yellow bristles on inner surface.

**Female.** Unknown.

#### Distribution.

China (Tibet).

#### Remarks.

The new species belongs to *D.fenestratus* group. It looks like *D.flexus*, but can be separated from the latter by the following features of males: the weak acute ventral process near extreme base of It_2_, the swollen apex of It_2_, the row of erect dense strong long posterior, and the anterior ventral bristles on apex of TII.

#### Etymology.

The name of new species refers to the strongly thickened legs.

### ﻿ Diostracus translucidus sp. nov.

Taxon classificationAnimaliaDipteraDolichopodidae

C76C0D85-8833-52BF-AF62-A2488071A1DE

https://zoobank.org/19498122-29A8-4F0E-8ADB-9318C2FD6101

Figs 25 , 26 , 27 , 28 , 29 , 30 , 31E , 32D

#### Type material.

***Holotype***: China • ♂, Tibet, Nyingchi, Medog, 80 k, 2013. IX. 13, leg. Gang Yao. ***Paratypes***: • 2 ♀♀, same data as for holotype.

#### Diagnosis.

MSSC: palpus normal, not reaching apex of proboscis. Scutellum with pair of sc and four or five pairs of marginal hairs; CI with row of anterior hairs and two strong recurved spines at extreme apex; FI with a deep hollow at base; wing indistinctly tinged grayish; FII with row of dense ad on apical 2/5; wing apically with three translucent windows between vein C, vein R_2+3_, vein R_4+5_, and vein M_1_.

#### Description.

**Figure 25.**
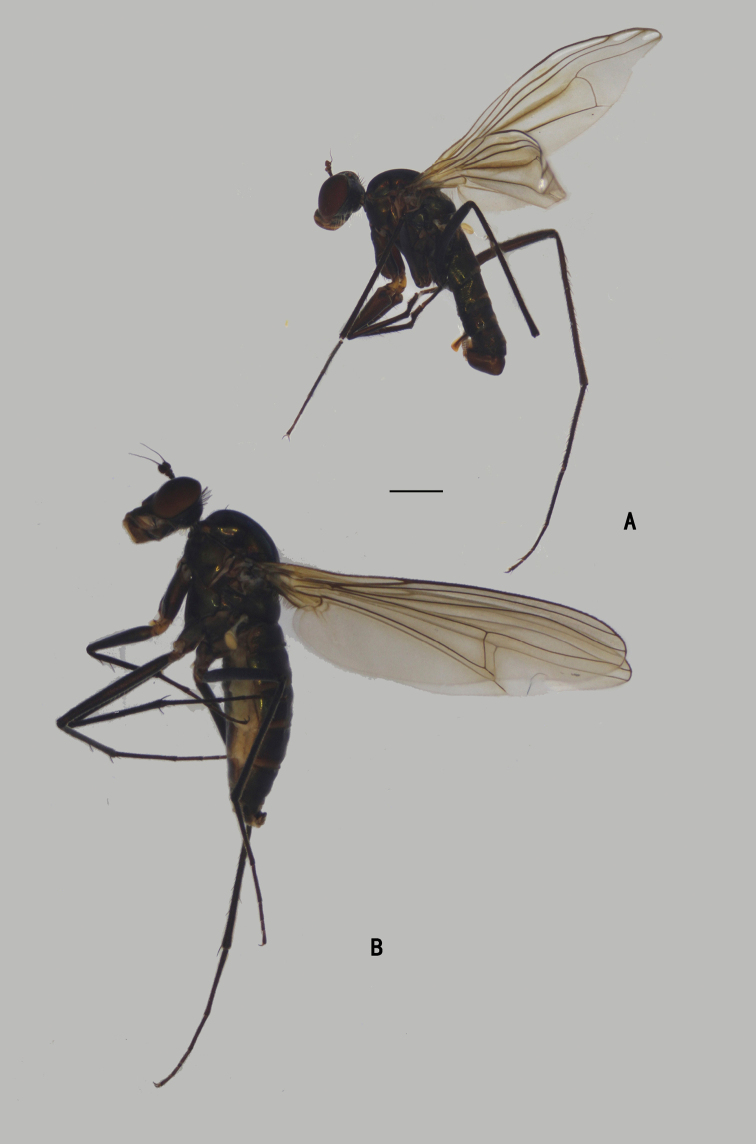
*D.translucidus***A** male; lateral view **B** female, lateral view. Scale bar: 1 mm.

**Male** (Fig. 25A). Body length 5.8 mm; wing length 6.0 mm.

**Figure 26.**
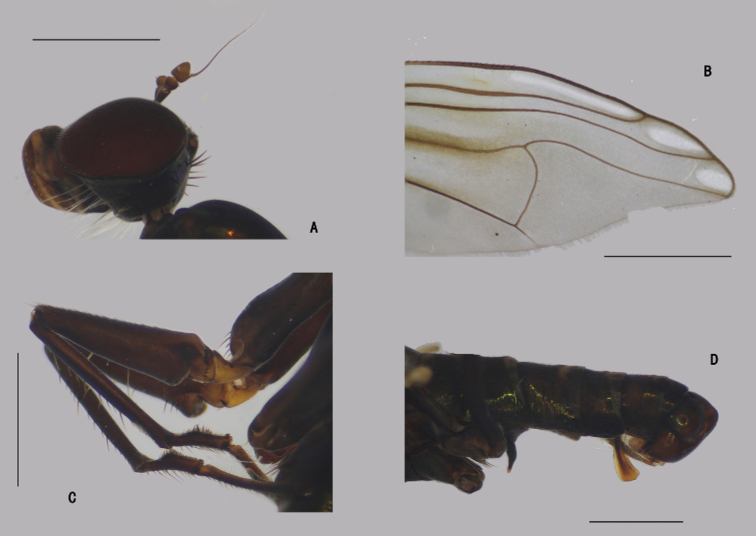
*D.translucidus* male **A** head, lateral view **B** apex of wing **C**LI, anterior view **D** abdomen, lateral view. Scale bars: 1 mm.

***Head*** (Fig. 26A) dark metallic green with pale gray pollinosity. Eyes separated; face widened towards clypeus. Hairs and bristles on head black; lower postocular bristles including posteroventral hairs pale. Ocellar tubercle distinct, with pair of oc (lost), without posterior hairs; vt rather short, 0.2 × as long as pvt. Antenna brownish black; scape prolonged, without dorsal bristle; first flagellomere subtriangular, 1.3 × longer than wide; arista apicodorsal, 4.8 × as long as first flagellomere, nearly bare. Proboscis huge and brown, apex blackish with pale hairs; palpus lobate, not reaching apex of proboscis, without distinctive bristle.

***Thorax*** dark metallic green with pale gray pollinosity; mesoscutum with pair of dark brown longitudinal stripe. Hairs and bristles on thorax black; six mostly hair-like dc except posterior most one dc longest and thick; acr absent; one h, one ph, one npl, one sa, one psa; scutellum with pair of sc (lost) and four or five pairs of marginal hairs (MSSC). Postnotum well developed, and convex. Propleuron with two or three sparse short pale hairs on lower portion.

***Legs*** nearly entirely black except fore trochanter dark yellow, mid- and hind trochanters brownish black; claws prolonged (MSSC), empodium and pulvilli present. Hairs and bristles on legs black. CI depressed laterally, without distinctive bristle, but with row of anterior hairs and two strong recurved spines at extreme apex (MSSC); CII and CIII with clusters of anterior bristles at extreme apex. FI distinctly thickened, with a deep hollow at base, and row of three or four curved ventral spines and row of dense anterior bristles long the edge of the hollow (MSSC), basal 2/3 with row of four long yellow av (nearly as long as FI depth) (MSSC), apically with long thin pale hairs (MSSC) (Fig. 26C); TI slightly thickened, with row of ad along whole length, ventral surface nearly bare (MSSC); It_1_ somewhat thicken, with row of long ad and pd along whole length (nearly as long as It_1_ depth), apical half flattened ventrally with two rows of short curved spines and rows of bristles along the edge, apically with a strong curved pv spine (MSSC) (Fig. 26C); It_2_ with row of long curved anteroventral hairs along the whole length, apical half with two rows of long posterior bristles (MSSC); It_3_ elongated, base and apex somewhat swollen, nearly bare, except with row of three or four posteroventral hairs at base and four or five dorsal bristles at apex (MSSC); It_4_ with long ventral bristles apically (MSSC). FII with row of dense ad on apical 2/5 (MSSC); TII with two ad, 8 pd. FIII without outstanding bristle; TIII with row of long thin ad and long erect thin pd; IIIt_1_ with row of thin ad long whole length. Relative lengths of tibia and five tarsomeres: LI 5.3: 1.2: 2.2: 2.6: 0.4: 0.7; LII 7.3: 5.0: 2.4: 1.2: 0.6: 0.8; LIII 8.5: 4.8: 3.4: 1.6: 0.8: 0.8.

***Wing*** (Fig. 26B) hyaline, indistinctly tinged grayish, apically with three translucent windows between vein C, vein R_2+3_, vein R_4+5_, vein M_1_ (MSSC); vein M with brown strip on middle section (MSSC); veins dark brown; crossvein m-cu somewhat curved. Squama yellow with yellow hairs. Halter brown.

**Figure 27.**
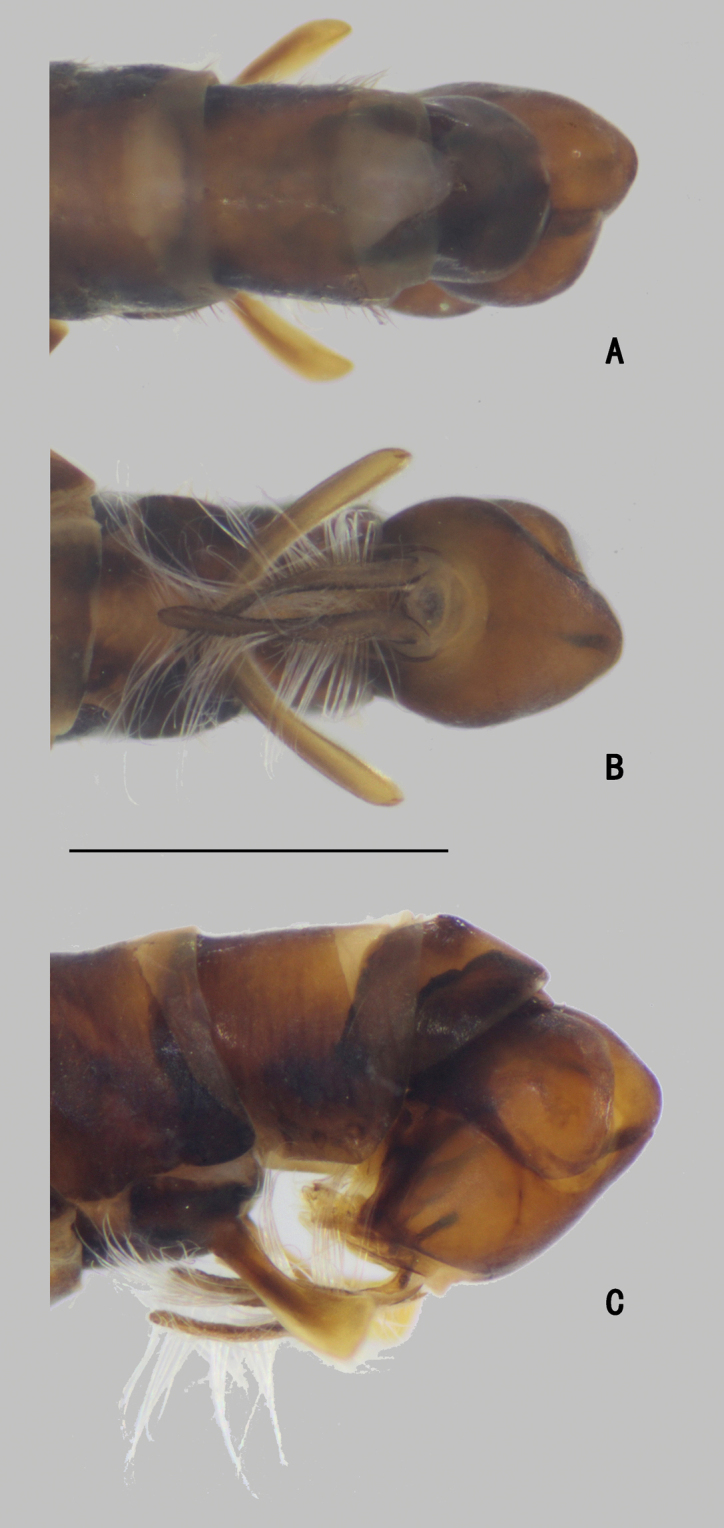
*D.translucidus* male, apex of abdomen **A** dorsal view **B** ventral view **C** lateral view. Scale bar: 1 mm.

**Figure 28.**
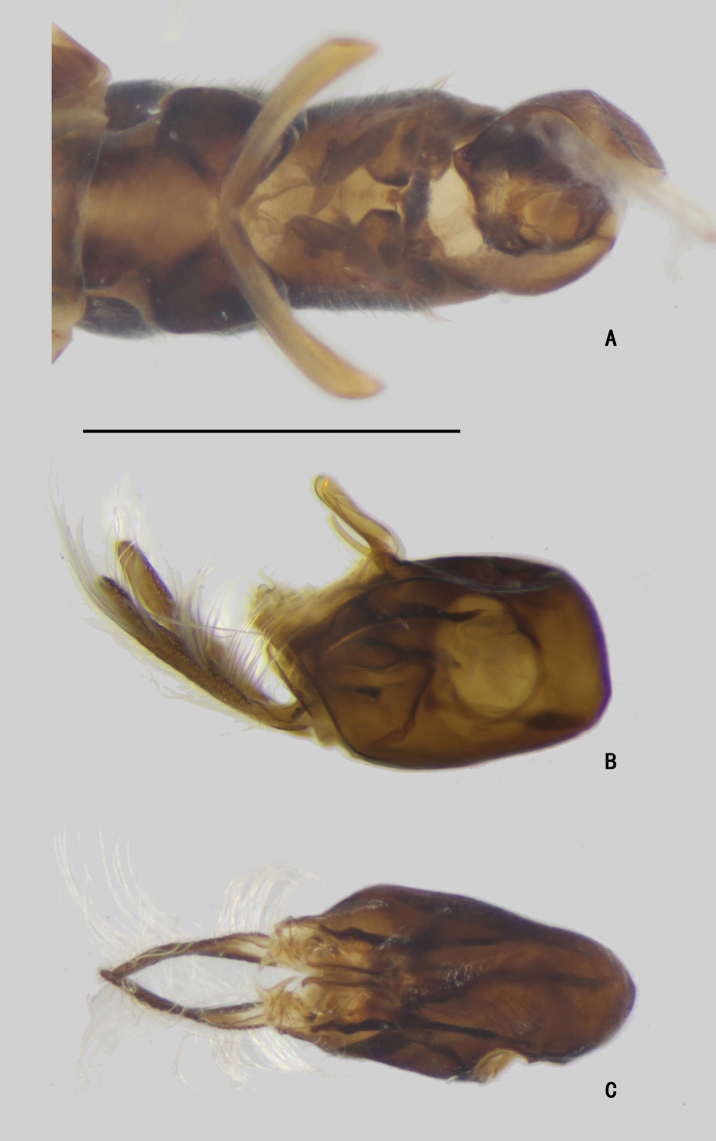
*D.translucidus* male **A** abdominal Sternite IV and V, male genitalia removed, ventral view **B** male genitalia, lateral view **C** male genitalia, ventral view. Scale bar: 1 mm.

**Figure 29.**
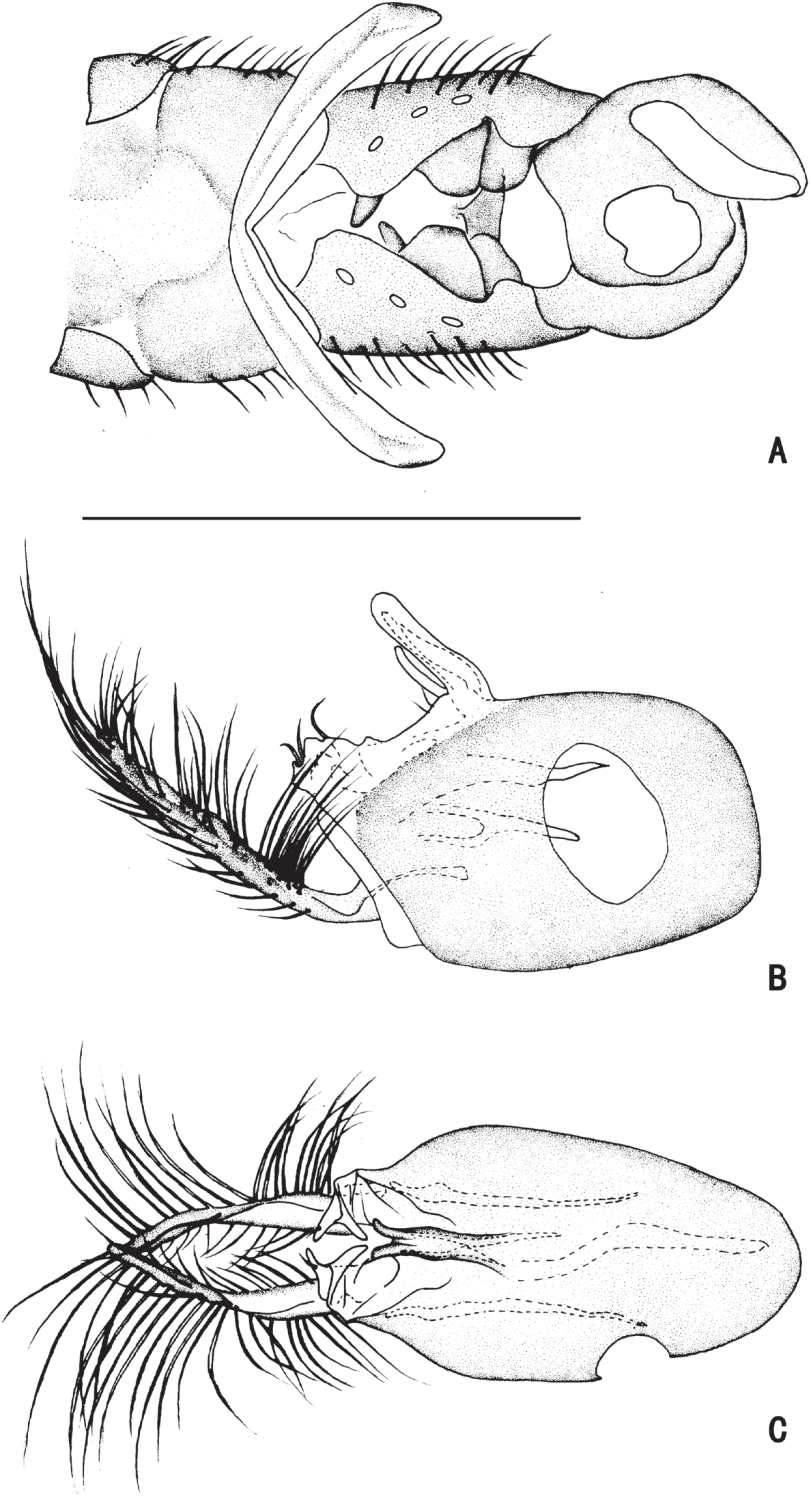
*D.translucidus* male **A** abdominal Sternite IV and V, male genitalia removed, ventral view **B** male genitalia, lateral view **C** male genitalia, ventral view. Scale bar: 1 mm.

***Abdomen*** (Fig. 26D) nearly as long as head and thorax combined, dark metallic green with pale gray pollinosity. Abdomen with sparse pale pubescence. Tergites II–V with triangular hyaline area on posterior margin (MSSC); Sternite II medially with a digitiform anterior process (MSSC) (Fig. 26D), Sternite IV with a pair of long brown band-like sclerites (Figs 27, 28A, 29A); Sternite V longer than Sternite IV, split into pair of sclerites (Figs 27, 28A, 29A). Hypandrium not distinctly swollen.

***Male genitalia*** (Figs 28B, C, 29B, C): Epandrium slightly longer than wide. Epandrial lobe long, wavy, band-like, with an acute basal process, one short bristle at tip. Surstylus thick, lamellated, with two short spines. Hypandrium short thick, apically with a shallow, V-shaped apical incision. Cercus rather short (1/3 as long as epandrium length), spoon-shaped, with dark yellow hairs on outer surface, apical ones long (nearly as long as cercus length), and subapically with group of dense erect dark yellow bristles on inner surface.

**Female** (Figs 25B, 31E). Body length 7.4–7.6 mm; wing length 7.8–8.0 mm. Same as male, except MSSC. FI without distinct bristles; TI with three ad, two pd; FII with two ad; TII with one ad, one pd, apically with three bristles; FIII bare; TIII with four ad, apically with two bristles. Wing (Fig. 32D) hyaline, indistinctly tinged grayish, crossvein m-cu straight, forming acute angle with CuA_1_.

**Figure 30.**
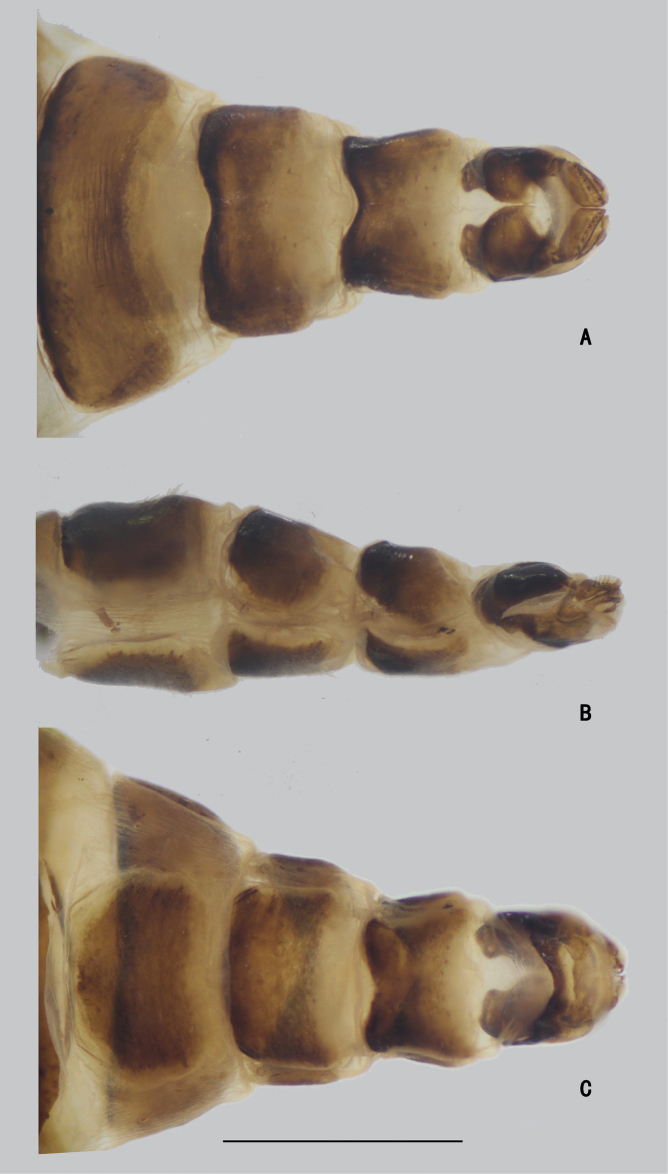
*D.translucidus* female, abdomen **A** dorsal view **B** lateral view **C** ventral view. Scale bar: 1 mm.

***Female genitalia*** (Fig. 30): Abdominal segments VII and VIII slender; tergite VIII divided into two sclerites; epiproct split into pair of hemitergites, apically with row of eight strong curved spines; hypoproct semicircular; dorsal lobes of cercus somewhat elongated, with dark yellow bristles; ventral lobes of cercus short, apex rounded.

**Figure 31.**
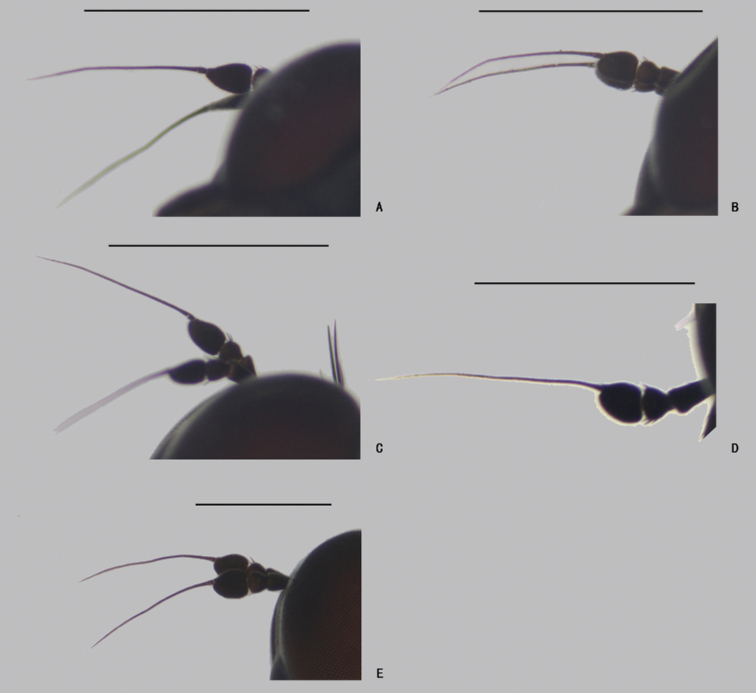
Female antenna, lateral view **A***D.acutatus*, show apical arista **B***D.acutatus*, show subapical arista **C***D.fasciculatus***D***D.polytrichus***E***D.translucidus*. Scale bars: 1 mm.

**Figure 32.**
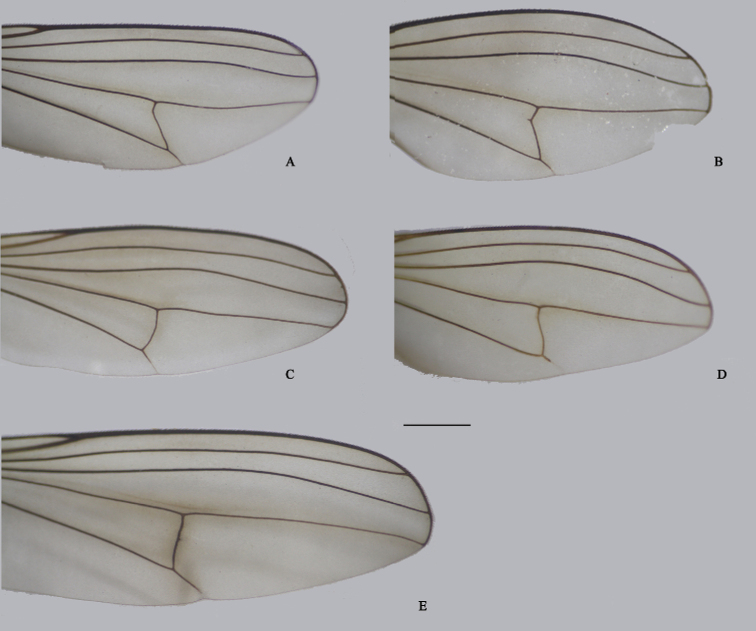
Apex of female wing **A, B***D.acutatus***C***D.fasciculatus***D***D.translucidus***E***D.polytrichus*. Scale bar: 1 mm.

#### Distribution.

China (Tibet).

#### Remarks.

The new species is unique. It has prolonged scapes, small palpus, and convex postnotum. But the huge proboscis, stout body and specialized structures of legs indicate that the new species belongs to *Diostracus*.

#### Etymology.

New species name refers to the translucent windows on male wing.

## ﻿Discussion

**Figure 33.**
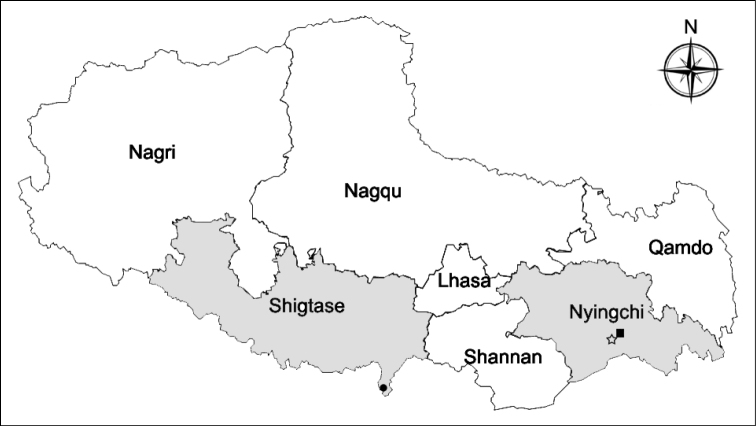
Distribution of new *Diostracus* species of Tibet: black circle: *D.fasciculatus*, *D.laetus*, *D.polytrichus*, and *D.strenus*; black square: *D.translucidus*; black star: *D.concavus*.

Including the species described in this work, the number of worldwide species of *Diostracus* has increased to 107, of which nine species occur in Tibet (Zhu 2006; Zhu et al. 2007a, b; Grichanov 2013, 2015, 2017; Pusch 2014; Wang et al. 2015). The six new species of *Diostracus* were found in Nyingchi and Shigatse areas of Tibet. Most of them were found at Yatung in Shigatse, which is located on the southern slope of the Himalaya Mountains (Fig. 33). As well as these two sites, we performed a five-year survey in Lhasa, Nyingchi, Shannan, and Qamdo, but there were no *Diostracus* found, although it does not mean *Diostracus* only occur in Nyingchi and Shigatse in Tibet. Due to the special habitat of the genus, it can only be collected with sweep nets after finding the flies using the naked eyes. Malaise traps collect the majority of insects in Tibet, as its environment is often too complex to walk through, but no *Diostracus* were found in them during these surveys.

*Diostracus* in the Oriental realm shows great diversity in MSSC, especially in modified FI and wing. Complex structures of FI are usually associated with modified wing. The most bizarre MSSC was shown in *D.fenestratus* group, with It_1_ distinctly shortened, almost triangular in shape, expanded portion concaved, and It_2_ sinuous, with a basal denticle (Saigusa 1984). The wing in this group is always ornamented, crossvein m-cu either in S-shape or running posteriorly parallel to M_1_. This species group includes 14 species all of which are distributed in the Himalayan Mountains. The species groups with partially thickened It_1_ and modified wings (mainly the *D.unisetosus* group) and the species groups with simple FI and modified wings (with a jet-black nodule besides crossvein m-cu, mainly the *D.unipunctatus* group) are distributed also mainly in the Himalayan Mountains. The species groups with partially thickened It_1_ and simple wings, mainly the *D.nebulosus* group, are distributed in the Himalayan Mountains and the Chinese mainland. Finally, the species groups with simple FI and simple wing is found in the Chinese mainland and Taiwan. Himalayan Mountains is the diversity center for the genus *Diostracus*, and species in this area show great diversity (53 species out of 107) and abundant specialized characters. The species in the Chinese mainland and Taiwan usually have low diversity (20 species of 107) and simple structures.

Besides the morphological characters, mitochondrial COI genes of females have also been sequenced to pair them with males. As a result, some females could not be matched to males, and the characters of these females are obviously different from known species found in our investigation. Therefore, we believe that more new species will be discovered in the Himalayan region in the future.

## Supplementary Material

XML Treatment for Diostracus

XML Treatment for Diostracus acutatus

XML Treatment for Diostracus concavus

XML Treatment for Diostracus fasciculatus

XML Treatment for Diostracus laetus

XML Treatment for Diostracus polytrichus

XML Treatment for Diostracus strenus

XML Treatment for Diostracus translucidus
